# Magnetic, EPR, and Computational Insights into ∼*D*_6*h*_ Tb(III) and Gd(III) Complexes

**DOI:** 10.1021/acs.inorgchem.6c01628

**Published:** 2026-08-07

**Authors:** Jishnu Gangadharan, Adam Brookfield, Tanu Sharma, Ethan Lowe, Claire Wilson, Angelos B. Canaj, Gopalan Rajaraman, David Collison, Mark Murrie

**Affiliations:** † School of Chemistry, 577311 University of Glasgow, University Avenue, Glasgow G12 8QQ, U.K.; ‡ Department of Chemistry, 5292 The University of Manchester, Oxford Road, Manchester M13 9PL, U.K.; § Department of Chemistry, 233704 Indian Institute of Technology Bombay, Powai, Mumbai, Maharashtra 400076, India

## Abstract

Lanthanide complexes continue to gain significant interest in molecular quantum science due to their rich electronic structures, which can be engineered to support protected spin states relevant for quantum technologies. Carefully tailored ligand fields can give rise to avoided level crossings between crystal-field perturbed states, which may reduce sensitivity to magnetic field fluctuations. Motivated by this, we investigate an axially compressed *pseudo*-*D*_6*h*_ symmetry air-stable macrocyclic Tb­(III) complex [Tb^III^(L^N6^)­(Ph_3_SiO)_2_]^+^ using *ab initio* calculations, magnetic and EPR measurements. The axially compressed, *pseudo*-*D*_6*h*_ symmetry imposes a ground state that is predominantly composed of *m*_*J*_ = ±6 for the oblate *J* = 6 Tb­(III) ion, with small admixtures from lower |*m*_*J*_⟩ components. We investigate the hexagonal vs nonhexagonal transverse crystal field contributions to the tunneling gap, calculating different models by selecting from the *ab initio* crystal field parameters. Our calculations predict a small tunneling gap, ≈1 GHz, and we test this prediction experimentally using multifrequency EPR on La­(III)-diluted powder samples. Our EPR results are consistent with the possibility of a low-energy (several GHz) tunneling gap. The Gd­(III) analog [Gd^III^(L^N6^)­(Ph_3_SiO)_2_]^+^ provides an orbital-quenched comparison, and its magnetic data and EPR spectra are well described using the axial zero-field splitting parameter *D*, demonstrating experimentally the largely axial ligand field in this *pseudo*-*D*_6*h*_ environment.

## Graphical abstract

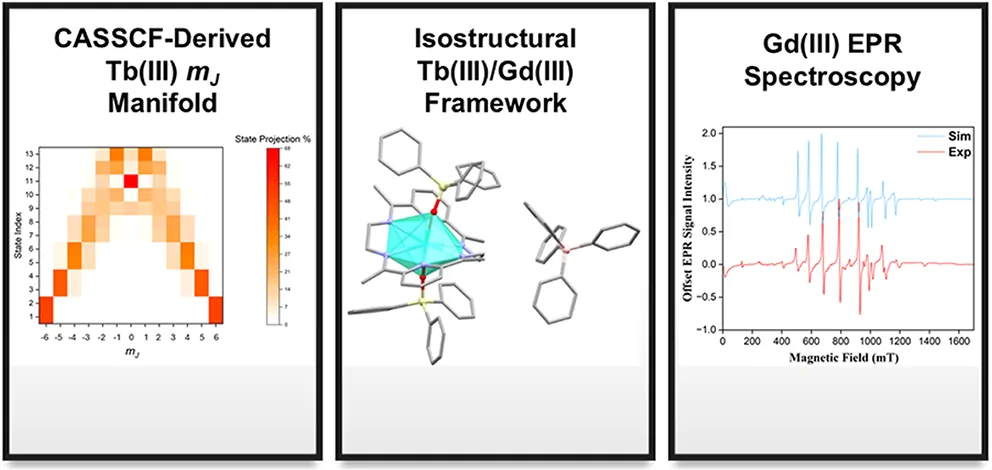

## Introduction

Lanthanide coordination complexes have attracted significant attention due to their distinctive electronic properties, making them promising candidates for applications in quantum information processing, communications and high-density data storage.^1–5^ Beyond their role as single-molecule magnets (SMMs),^6–9^ these systems are being explored as molecular qubits.^10^ Furthermore, the magnetic states associated with lanthanide ions, such as Ho­(III), are excellent hosts for spin clock transitions (SCTs).^11^ SCTs occur at avoided Zeeman level crossings between a pair of nondegenerate energy states (perturbed by crystal field interactions) that enhance coherence by fortifying qubits against magnetic noise.^12,13^ Achieving SCT behavior requires precise control over the ligand field to stabilize the dominant ± *m*_*J*_ ground doublet, while selectively enabling higher-order mixing terms that open a symmetry-protected tunneling gap.^10,14,15^

In this context, axially compressed *pseudo*-*D*_6*h*_ symmetric lanthanide complexes e.g., containing the L^N6^ (C_22_H_26_N_6_) macrocyclic equatorial ligand (see Scheme 1) are appealing as they allow coordination of strong axial ligands, while maintaining excellent thermal and air stability. First, the combination of short axial (O) and long equatorial (N) bonds should stabilize an *m*_*J*_ = ±6 quasi-doublet ground state for the oblate *J* = 6 Tb­(III) ion. Second, the balance between axial purity and mixing via the transverse crystal field should generate a tunneling gap. Previously, we have shown that complexes such as [Dy^III^(L^N6^)­(Ph_3_SiO)_2_]^+^ enforce strong axiality and yield large SMM anisotropy barriers, highlighting the value of symmetry control in lanthanide macrocyclic complexes.^16^ Our aim is to confirm that the strongly axial ligand field stabilizes an *m*_*J*_ = ±6 quasi-doublet ground state for Tb­(III),^17^ while introducing the transverse mixing that creates a tunneling gap.^18^

**Scheme 1:**
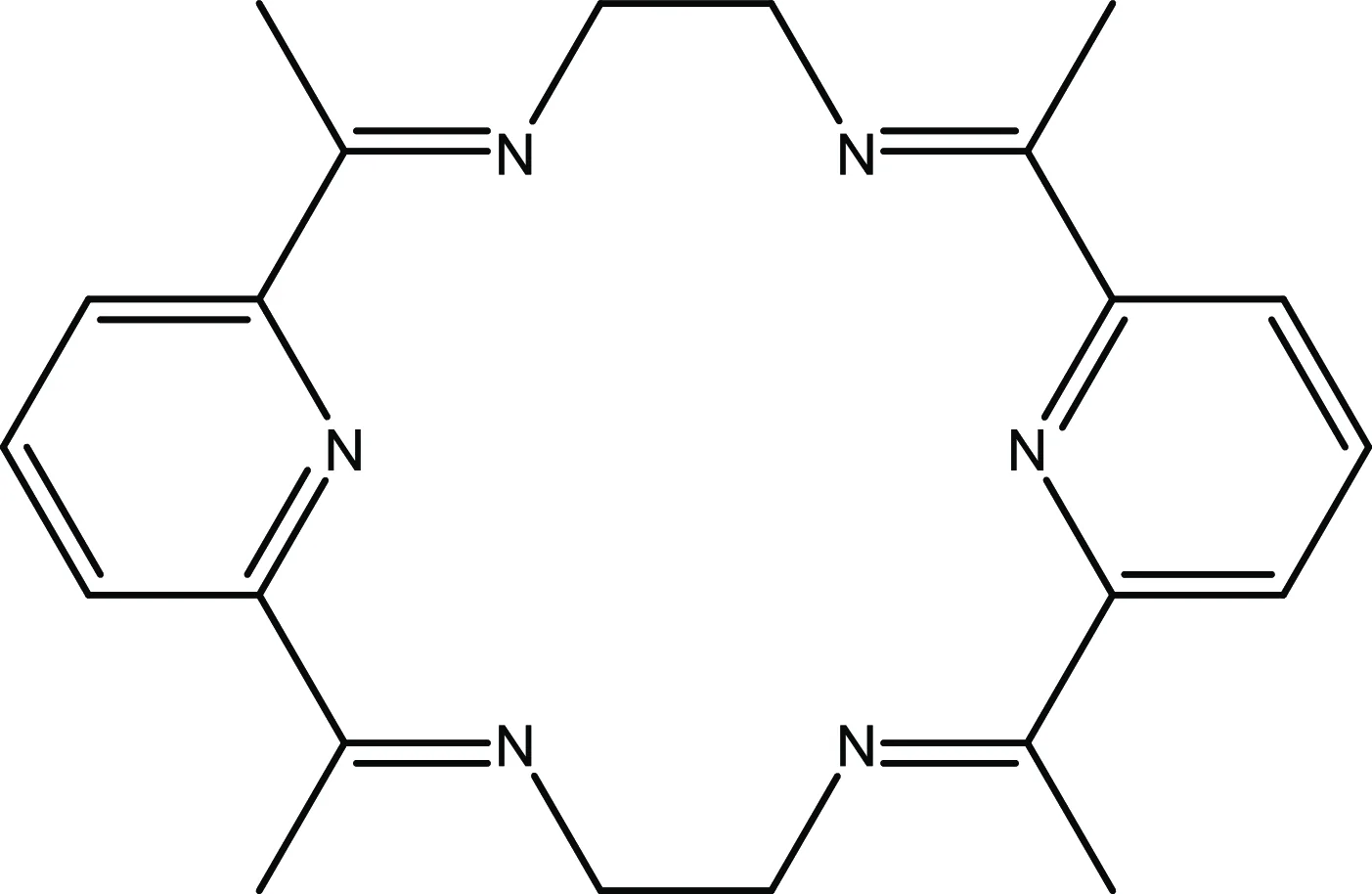
Structure of the L^N6^ Ligand, C_22_H_26_N_6_

Electron paramagnetic resonance (EPR) spectroscopy is ideally suited here, because it directly measures the energy splittings imposed by the crystal field.^19,20^ Yet, no EPR studies of *pseudo*-*D*_6*h*_ Tb­(III) macrocyclic complexes have been reported. Herein, we show that *ab initio* calculations predict a tunnel splitting of ≈1 GHz in [Tb^III^(L^N6^)­(Ph_3_SiO)_2_]^+^. Accordingly, we present the EPR study of a *pseudo*-*D*_6*h*_ Tb­(III) macrocyclic complex to investigate its electronic structure. We have also synthesized and studied a Gd­(III) analog of the Tb­(III) complex. Given that the EPR spectra of Gd­(III) can be reliably described using an effective spin Hamiltonian framework, this analog serves as a valuable reference to compare the crystal field with the Tb­(III) system, particularly given the absence of ground state orbital angular momentum in Gd­(III).^17,21–23^

## Experimental Section

All reagents were used as received without any further purification.

*Synthesis of [Tb* ^*III*^ *L* ^*N6*^ *(Ph*_*3*_ *SiO)*_*2*_ *]­(BPh*_*4*_ *) (**1**) {L* ^*N6*^ *= macrocyclic equatorial Schiff base ligand formed from 2,6-diacetylpyridine and ethylenediamine, see* Scheme 1}. TbCl_3_·6H_2_O (0.093 g, 0.25 mmol) and 2,6-diacetylpyridine (0.082 g, 0.5 mmol) were dissolved in 10 mL of methanol. Ethylenediamine (0.033 mL, 0.5 mmol) was then added, resulting in a yellow solution. This mixture was refluxed for 12 h and the solvent was removed under vacuum, giving a brown oil. This oil was dissolved in 10 mL of dichloromethane and stirred for 10 min. The solution was then added to a 3 mL solution of sodium tetraphenylborate (0.017 g, 0.05 mmol) and triphenylsilanol (0.055 g, 0.2 mmol) in deionized water. A brown suspension with two distinct phases was formed: the upper aqueous layer and the lower CH_2_Cl_2_ layer. The suspension was heated to 80 °C for 40 min with continuous stirring. The CH_2_Cl_2_ layer was then separated, filtered and used in vapor diffusion using Et_2_O. Yellow needle-like crystals of **1** were obtained over the course of 3 to 4 days. Elemental Analysis calcd. (found) for C_82_H_76_BN_6_O_2_Si_2_Tb: C 70.18 (69.98), H 5.46 (5.45), N 5.99 (6.10) %. Selected IR Data *ṽ* (cm^–1^): 3052, 2997, 1643, 1586, 1479, 1424, 1372, 1252, 1195, 1106, 968, 815, 736, 697, 609.

*Synthesis of [Gd* ^*III*^ *L* ^*N6*^ *(Ph*_*3*_ *SiO)*_*2*_ *]­(BPh*_*4*_ *)* *(**2**)*. The same procedure as reported above for **1**, but using GdCl_3_·6H_2_O (0.093 g, 0.25 mmol) in place of TbCl_3_·6H_2_O, was followed for the synthesis of **2**, which appears as yellow column-like crystals within 3 to 4 days. Elemental analysis calcd. (found) for C_82_H_76_BGdN_6_O_2_Si_2_: C 70.26 (70.37), Η 5.46 (5.56), Ν 6.00 (6.22) %. Selected IR Data *ṽ* (cm^–1^): 3053, 2998, 1641, 1586, 1479, 1427, 1377, 1250, 1195, 1108, 968, 812, 734, 698, 639.

*Synthesis of [La* ^*III*^ *L* ^*N6*^ *(Ph*_*3*_ *SiO)*_*2*_ *]­(BPh*_*4*_ *)* *(**3**)*. The same procedure as reported above for **1**, but using LaCl_3_·7H_2_O (0.092 g, 0.25 mmol) in place of TbCl_3_·6H_2_O, was followed for the synthesis of **3**, which appears as yellow block-like crystals within 3 to 4 days. Elemental analysis calcd. (found) for C_82_H_76_BLaN_6_O_2_Si_2_·H_2_O: C 70.28 (70.45), Η 5.61 (5.57), Ν 6.00 (6.14) %. Selected IR Data *ṽ* (cm^–1^): 3056, 2951, 2354, 1946, 1635, 1479, 1372, 1287, 1191, 1104, 998, 937, 810, 699, 623.

*Synthetic strategy for 1@La, 1′@La and 2@La.* For the synthesis of the diluted compounds, **1@La**, **1′@La** and **2@La**, the same procedure is applicable by using a combination of TbCl_3_·6H_2_O and LaCl_3_·6H_2_O or GdCl_3_·6H_2_O and LaCl_3_·6H_2_O, respectively. To achieve a 6% Tb dilution of **1@La**, TbCl_3_·6H_2_O (0.025 mmol, 0.0093 g) and LaCl_3_·6H_2_O (0.225 mmol, 0.084 g) were used whereas for a 0.7% Tb dilution of **1′@La**, TbCl_3_·6H_2_O (0.0025 mmol, 0.00093 g) and LaCl_3_·6H_2_O (0.248 mmol, 0.092 g) were used. Furthermore, to achieve a 8.8% Gd dilution of **2@La**, GdCl_3_·6H_2_O (0.025 mmol, 0.0093 g) and LaCl_3_·6H_2_O (0.225 mmol, 0.084 g) were used.

### Theoretical Calculations

To rationalize the magnetic properties of **1**, *ab initio* calculations were performed using the MOLCAS 8.2 suite.^24–26^ The ANO-RCC-VTZP basis set was used for Tb, while the ANO-RCC-VDZP basis set was applied to rest of the atoms. Scalar relativistic effects were incorporated via the Douglas-Kroll-Hess (DKH) Hamiltonian. Initial guess orbitals were generated using DFT calculations, followed by CASSCF calculations with CAS­(8,7) for Tb^III^. The spin-free states from CASSCF were then mixed using the RASSI-SO module,^27^ yielding 7 septets, 140 quintets, and 195 triplets. SINGLE_ANISO was employed to compute *g*-tensors, magnetic anisotropy, crystal field parameters and the tunnel splitting.

## Results and Discussion

### Structure of **1**

Compound **1** crystallizes in the monoclinic space group *P*2_1_/*c* (Table S1). The asymmetric unit consists of a [Tb^III^L^N6^(Ph_3_SiO)_2_]^+^ complex cation with a BPh_4_^–^ anion (see Figure 1). The Tb­(III) center exhibits an approximate hexagonal bipyramidal geometry ∼*D*_6*h*_, in the first coordination sphere, as confirmed by continuous SHAPE measures analysis (CShMs) with a score of 2.029 (Table S2) where a score of 0 indicates a perfect polyhedron.^28^ The Tb­(III) ion is equatorially coordinated by the six nitrogen atoms of the L^N6^ macrocycle with two Ph_3_SiO^–^ ligands in the axial positions. The two Ph_3_SiO^–^ ligands in the axial positions provide short Tb–O bonds of 2.1497(8) and 2.1659(8) Å (see Table S3). In addition, the axial O–Tb–O bond angle is 170.74(3)°. The Tb–N bond distances and N–Tb–N bond angles in the equatorial plane fall in the range of 2.6202(9)–2.6733(9) Å and 59.58(3)–63.60(3)°, respectively (see Table S4). Comparing **1** with our previously published Dy­(III) complex reveals different packing arrangements (Figure S1 and S2).^16^ The shortest intermolecular Tb···Tb distance is 11.602 Å (Figure S3) while the shortest Dy···Dy distance is 10.896 Å. To minimize the effect of magnetic spin–spin dipolar interactions between Tb···Tb, compound **1** was diluted in two different ratios (6% and 0.7% Tb), yielding **1@La** and **1′@La** (Figure S4 and S5). The La­(III) analog crystallizes in the same space group as **1**, with similar unit cell parameters (see below and Table S1).

**Figure 1:**
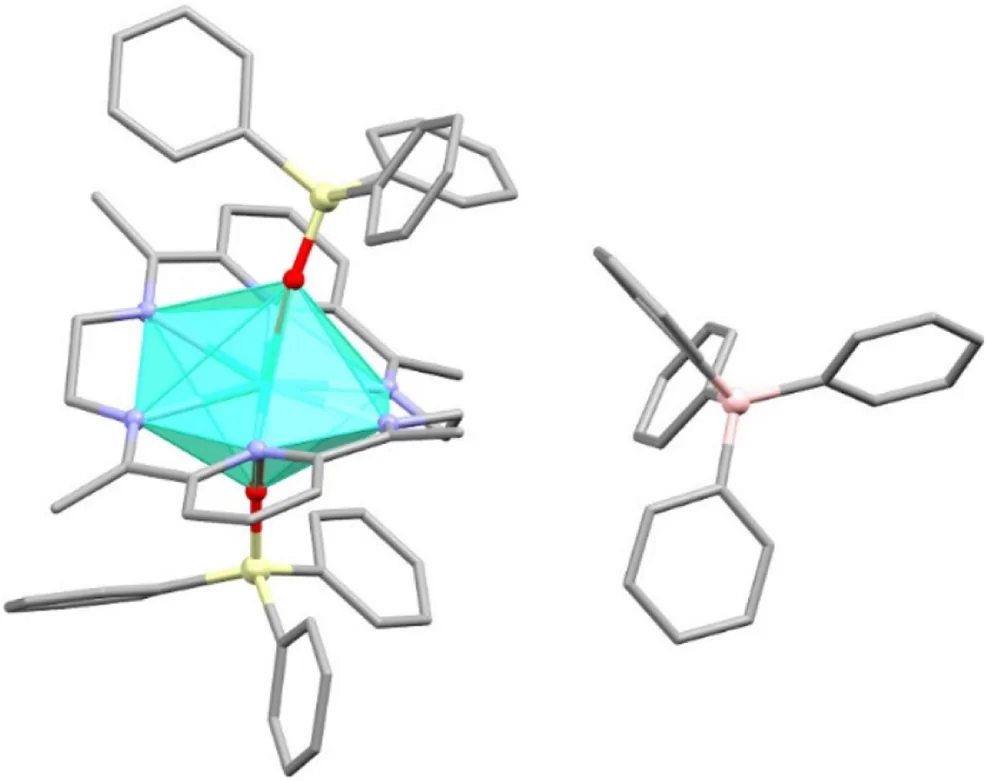
Structure of **1** highlighting the axially compressed ∼hexagonal bipyramidal geometry of the Tb­(III) ion. Tb (cyan); C (gray); N (blue); O (red); Si (yellow); B (pink); and H omitted for clarity.

### Structure of **2**

Compound **2** crystallizes in the monoclinic space group *P*2_1_/*c* and is isostructural with compound **1** (see Figure S6). Due to its larger ionic radius, the Gd­(III) ion achieves more effective encapsulation within the L^N6^ macrocyclic framework,^29,30^ resulting in slightly reduced distortion of the first coordination sphere (CShMs score of 1.978 for a hexagonal bipyramid compared to 2.029 for **1**, see Table S2 and Figures S7 and S8). The two Ph_3_SiO^–^ ligands in the axial positions provide short Gd–O bonds of 2.1902(18) and 2.1750(18) Å (see Table S3). In addition, the axial O–Gd–O bond angle is 170.69(7)°. The Gd–N bond distances and N–Gd–N bond angles in the equatorial plane fall in the range of 2.626(2)–2.678(2) Å and 59.52(7)–63.61(7)°, respectively (see Tables S3 and S4). The shortest Gd···Gd intermolecular distance is 11.617 Å (see Figure S9). To minimize magnetic dipolar interactions, compound **2** was also diluted to form **2@La**, with an 8.8% Gd to La doping ratio, ensuring consistency with the space group (see Table S1) and incorporating diamagnetic La­(III) between the Gd­(III) centers (see Figure S10).

### Structure of **3**

Compound **3** crystallizes in the monoclinic space group *P*2_1_ */c* and is isostructural with both **1** and **2** (Figure S11 and Table S1). The La­(III) ion, being larger than both Tb­(III) and Gd­(III), is encapsulated most effectively with least distortion as compared to both **1** and **2** (see CShM scores, Table S2). See ESI for the bond distances and angles (Table S3).

The phase purity of compounds **1**, **2** and **3** was confirmed by powder X-ray diffraction (see Figure S12).

### Magnetic Properties

Magnetic susceptibility measurements were carried out from 280 to 2 K for both compounds **1** and **2** in a static dc field of 1000 Oe (see Figures 2 and 3). Upon cooling, *χ_M_T* for **1** steadily decreases from 11.44 cm^3^ mol^–1^ K at 280 K reaching 10.77 cm^3^ mol^–1^ K at 100 K and 8.76 cm^3^ mol^–1^ K at 2 K, due to depopulation of the Stark levels (see Figure 2).^31–33^ The *χ_M_T* product for **1** at 280 K is slightly lower than the theoretical value (11.81 cm^3^ mol^–1^ K), which can be related to ligand field effects.^34,35^ Magnetization experiments were also carried out for **1** with a variable dc field up to 7 T (Figure 2 inset). At 2 K, M = 4.87 Nβ, consistent with a highly anisotropic *m*_*J*_ = ±6 ground state.^36,37^ In compound **2**, *χ_M_T* at 280 K (7.89 cm^3^ mol^–1^ K) is consistent with the isotropic nature of Gd­(III) (*g* = 2, *χ_M_T*_calc_ = 7.88 cm^3^ mol^–1^ K) (see Figure 3).^19^ A small decrease in *χ_M_T* below 5 K may be indicative of a very small magnetic anisotropy.^16^ Magnetization experiments were carried out on **2** (Figure 3 inset). The observed magnetization (6.97 Nβ) at 2 K for compound **2** is very close to the theoretical M_sat_ (7.0 Nβ) which again can be attributed to the isotropic nature of Gd­(III).^17,38^ Hence, the *χ_M_T* *vs T* and magnetization vs. field data were fitted simultaneously (see Figure 3) using the Hamiltonian

**Figure 2:**
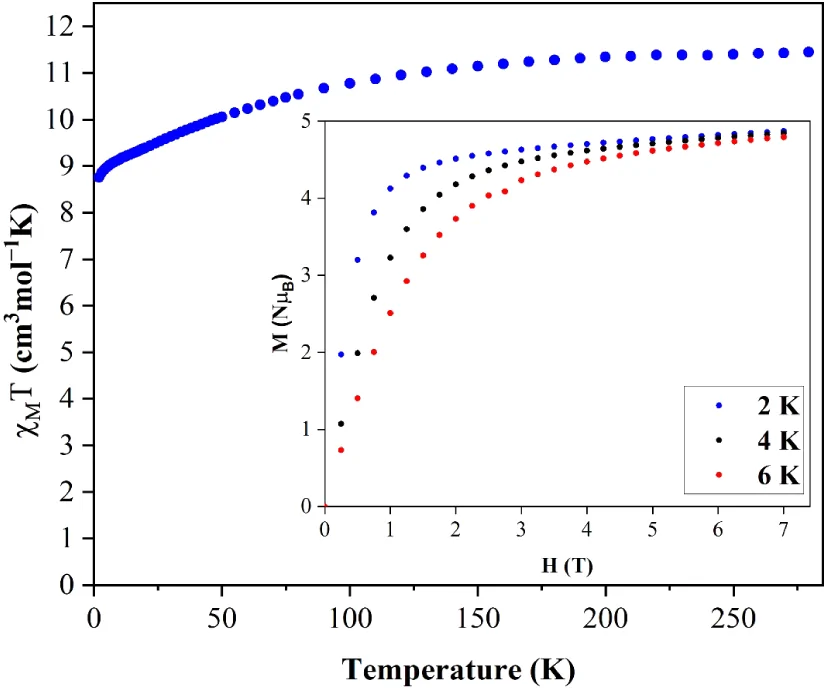
Temperature dependence of *χ_M_T* from 280 to 2 K for **1**. The variable-field magnetization of **1** at 2, 4, and 6 K (inset).

**Figure 3:**
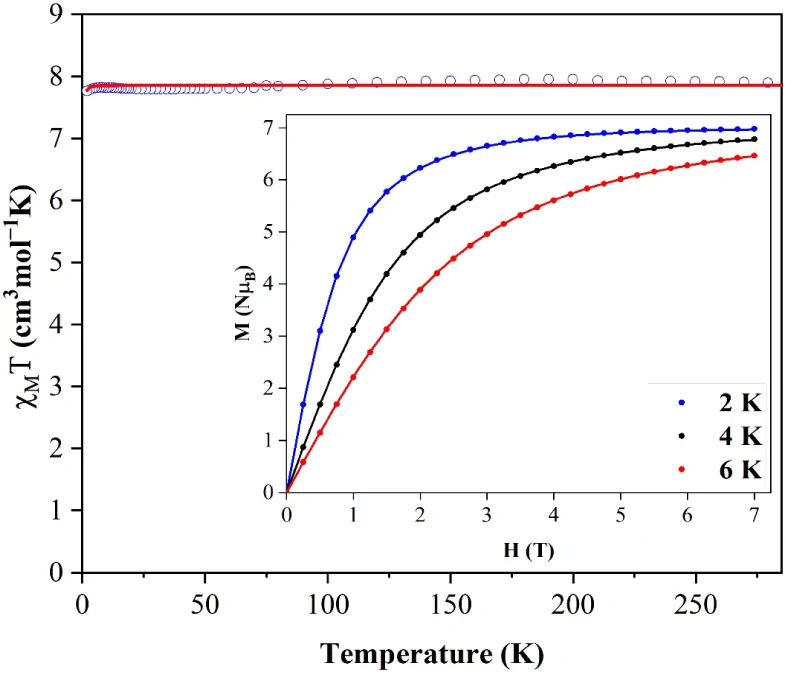
Temperature dependence of *χ_M_T* from 280 to 2 K for **2** (blue open circles). The variable-field magnetization of **2** at 2, 4, and 6 K (inset). For details of the fit, see text.

$Ĥ=D{Ŝ}_{z}^{2}+\mu_{B}\mathrm{B⃗}\mathrm{g⃡}Ŝ$ for **2** using PHI which gives *g* = 1.9969 ± 0.0002 and *D* = 0.131 cm^–1^ ± 0.002, where *μ_B_* is the Bohr magneton, B⃗ is the magnetic field, g⃡ is the *g*-matrix and *Ŝ* is the spin operator, respectively.^39^ The data can be fitted instead with a negative *D* parameter, but with a slightly larger residual. Both *g* and |*D*| obtained from the fit are consistent with those obtained from our EPR experiments (see EPR section).

### Theoretical Studies

To evaluate the splitting and mixing of the states, the crystal field parameters and tunnel splitting of compound **1**, *ab initio* CASSCF/RASSI-SO/SINGLE_ANISO calculations were performed. The axially compressed, *pseudo*-*D*_6*h*_ symmetry imposes a ground state that is largely composed of *m*_*J*_ = ±6 for the oblate *J* = 6 Tb­(III) ion. However, these calculations reveal that the ground state is not a perfectly pure *m*_*J*_ = ±6 doublet but contains small admixtures from lower |*m*_*J*_⟩ components (Table 1). Specifically, while the dominant contribution for **1** is 0.497 |+6⟩ + 0.497 |−6⟩, as expected due to the strong axial ligand field provided by the short Tb–O bonds, there are minor but non-negligible components of, in order of magnitude, |±4⟩, |±3⟩ and |0⟩, as well as further smaller components (see Table 1). This subtle mixing is crucial, as it introduces transverse anisotropy into what would otherwise be an ideal Ising-type ground state. Although the *g-*matrix values confirm strong axiality (*g*_*xx*_ = 0.0, *g*_*yy*_ = 0.0, *g*_*zz*_ = 17.9) the presence of small transverse terms explains the finite calculated tunnel splitting in the ground doublet for **1** (0.028 cm^–1^, ≈0.84 GHz) (Table 1 and Table S5). Upon removing the counteranion (modeled as **1**_wc_, see Tables S5 and S7) the calculated tunnel splitting increases from ≈0.84 GHz to ≈0.93 GHz, implying that even modest changes in the electrostatic environment can influence the tunneling gap. As the system progresses to higher excited states, the wave functions become increasingly mixed (Tables S6, S7), with state 2 dominated by |±5⟩ contributions and states 3–6 showing significant weight from |±4⟩, |±3⟩, |±2⟩, |±1⟩, and |0⟩. This evolution reduces *g*_*zz*_ steadily from 15.3 in state 2, to 2.2 in state 6, while the angles between the principal magnetic axes of the ground and excited states remain large (≈19–20°), highlighting strong misalignment of these axes and mixing.

**Table 1:** Calculated Energies, Composition of States with Full Numerical Precision, Tunnel Splitting and *g_zz_* of the Ground State *Pseudo-*Doublet in **1**

Compound	Energies (cm^–1^)	Composition of states	Tunnel splitting (cm^–1^)	Tunnel splitting (GHz)	*g*_*zz*_
**1**	0.000	0.497335 |+6⟩ + 0.497335 |−6⟩	0.028	0.84	17.9
		0.000005 |+5⟩ + 0.000005 |−5⟩			
		0.002358 |+4⟩ + 0.002358 |−4⟩			
		0.000189 |+3⟩ + 0.000189 |−3⟩			
		0.000006 |+2⟩ + 0.000006 |−2⟩			
		0.000058 |+1⟩ + 0.000058 |−1⟩			
		0.000103 |0⟩			
	0.028	0.497348 |+6⟩ + 0.497348 |−6⟩			
		0.000006 |+5⟩ + 0.000006 |−5⟩			
		0.002381 |+4⟩ + 0.002381 |−4⟩			
		0.000196 |+3⟩ + 0.000196 |−3⟩			
		0.000027 |+2⟩ + 0.000027 |−2⟩			
		0.000038 |+1⟩ + 0.000038 |−1⟩			
		0.000001 |0⟩			

The crystal field parameters $\left(B_{k}^{q}\right)$ are listed in Table S8 (*k* = 2, 4, 6) and Table S9 (*k* = 8, 10, 12). In perfect *D*_6*h*_ symmetry, the allowed terms for *k* ≤ 6 are $B_{2}^{0}$ , $B_{4}^{0}$ , $B_{6}^{0}$ and $B_{6}^{\pm 6}$ . However, in **1**, the symmetry is lower and further terms arise from the distorted *pseudo*-*D*_6*h*_ geometry i.e., the nonplanarity of the macrocyclic L^N6^ ring and the deviation from a perfect 180° axial O–Tb–O bond angle. Together these factors enforce transverse crystal field components. The consequence is a ground state that, while mainly axial, carries enough transverse admixture to generate a small tunnel splitting. This delicate balance between axial purity and transverse mixing is vital.

Engineering a match between the absolute |*m*_*J*_| value of the ground-state doublet and the rotational symmetry of the Ln­(III) coordination environment is an important factor for the appearance of a tunneling gap: for a given $B_{k}^{\pm q}{Ô}_{k}^{\pm q}$ with *q* ≠ 0, |*m*_*J*_⟩ can couple to |*m*_*J*_ ± *q*⟩. Following this argument, in perfect *D*_6*h*_ symmetry the transverse terms for *k* ≤ 6 are $B_{6}^{\pm 6}{Ô}_{6}^{\pm 6}$ , coupling |±6⟩ to |0⟩. Only one linear combination of |+6⟩ and |−6⟩ has a nonzero matrix element with |0⟩ and the ${Ô}_{6}^{\pm 6}$ operator lifts the degeneracy of the |±6⟩ doublet by selectively mixing one symmetry-adapted combination with |0⟩. This can be seen in the composition of states (Table 1) where |0⟩ appears only by looking to the sixth decimal place (i.e., effectively zero mixing) for the state at 0.028 cm^–1^. An interesting question is why the calculated gap is much smaller for **1** than in the, albeit structurally different, Ho­(III) ∼*C*_4*v*_ complex.^11^ In **1**, the calculated $B_{6}^{6}{Ô}_{6}^{6}$ term is around 20 times smaller than the leading calculated transverse $B_{4}^{4}{Ô}_{4}^{4}$ term in the Ho­(III) ∼*C*_4*v*_ complex. The composition of states shows that the level of admixing into the ground *pseudo*-doublet is smaller in **1** than for the Ho­(III) ∼*C*_4*v*_ complex, which is consistent with the much smaller calculated tunneling gap for **1**.

However, a more important question is whether $B_{6}^{\pm 6}{Ô}_{6}^{\pm 6}$ is a sufficient design descriptor here, where the symmetry is lower than perfect *D*_6*h*_. To explore this, we reconstructed the Tb­(III) crystal-field Hamiltonian, using the *ab initio* crystal field parameters for **1** and calculated the tunneling gap implemented in EasySpin.^40^ When considering only the *k* = 2 crystal field parameters there is no tunneling gap, but a small gap (0.02 GHz) opens for **1** upon adding the *k* = 4 parameters (Table S10). The *ab initio* calculated gap of 0.84 GHz is reproduced in EasySpin by including all higher rank crystal field parameters to *k* ≤ 12. However, reducing this to include only *k* ≤ 6 increases the calculated gap to ≈5.8 GHz (see Table S10), indicating that some higher rank terms of the crystal field are acting to reduce the gap.

Next, we compare the allowed hexagonal vs nonhexagonal transverse contributions for *k* ≤ 6 and for *k* ≤ 12 (see Table 2). This reveals that neither the *D*_6*h*_-allowed terms alone (for *q* = 0, ±6 and for *q* = 0, ±6, ±12) nor the other symmetry-breaking terms alone reproduce the *ab initio* gap exactly. However, the *D*_6*h*_ segment yields a splitting of 0.79 GHz (for *k* ≤ 12), which is ≈94% of the *ab initio* gap, although the complementary nonhexagonal segment generates a larger splitting of 3.31 GHz. When considering only *k* ≤ 6 (see Table 2) the *D*_6*h*_ sector gives a larger splitting of 7.18 GHz and the complementary nonhexagonal sector gives a smaller splitting of 0.73 GHz. Only when both sectors are included simultaneously (for *k* ≤ 12), i.e., the full Hamiltonian, is the *ab initio* calculated gap of 0.84 GHz recovered. Importantly, this analysis shows that the gap in **1** is not controlled by a single tunneling pathway and originates from competing contributions from different tunneling pathways. The observation of EPR spectra for **1** serves as confirmation of the presence of transverse interactions in the crystal field Hamiltonian (*vide infra*).

**Table 2:** Effects on the Calculated Tunnel Splitting in **1** Based on Symmetry-Restricted Models

Model	Tunnel splitting (GHz)
Hexagonal *model 1,* for *k* ≤ 6 ( $B_{2}^{0}$ , $B_{4}^{0}$ , $B_{6}^{0}$ , $B_{6}^{\pm 6}$ )	7.18
Non-hexagonal *model 1* (all terms for *k* ≤ 6 excluding $B_{6}^{\pm 6}$ )	0.73
Hexagonal *model 2,* for *k* ≤ 12 ( $B_{2}^{0}$ , $B_{4}^{0}$ , $B_{6}^{0}$ , $B_{8}^{0}$ , $B_{10}^{0}$ , $B_{12}^{0}$ , $B_{6}^{\pm 6}$ , $B_{8}^{\pm 6}$ , $B_{10}^{\pm 6}$ , $B_{12}^{\pm 6}$ , $B_{12}^{\pm 12}$ )	0.79
Non-hexagonal *model 2* (all terms for *k* ≤ 12 excluding $B_{6}^{\pm 6}$ , $B_{8}^{\pm 6}$ , $B_{10}^{\pm 6}$ , $B_{12}^{\pm 6}$ , $B_{12}^{\pm 12}$ )	3.31
Full Hamiltonian (*k* ≤ 12)	0.84
*Ab initio*	0.84

### EPR Analysis of 1

Among the non-Kramers lanthanide ions, Tb­(III) (4f^8^, ^7^F_6_, *S* = 3, *L* = 3, *J* = 6) and Tm­(III) (4f^12^, ^3^H_6_, *S* = 1, *L* = 5, *J* = 6) both possess a *J* = 6 ground multiplet, and extensive electron paramagnetic resonance (EPR) studies have been carried out on each. Since the present study focuses on Tb­(III), only previous results relevant to this ion are discussed below. Early theoretical analyses predicted that Tb­(III) would exhibit a singlet crystal field ground state,^41,42^ which would nominally render it EPR silent. However, subsequent studies revealed that mixing with the first excited crystal field level enables EPR transitions, giving rise to an effective spin-1/2 ground doublet with strong axial anisotropy (*g*_∥_ ≫ *g*_⊥_). The first-derivative X-band CW spectra of Tb­(III) are typically characterized by a dominant low-field feature (∼600 G) and near-zero slope at high fields (>5000 G). Signals often saturate at 4.2 K and weaken above 10 K, while pulsed and echo-detected spectra display richer structure due to hyperfine coupling and field-dependent relaxation.^43,44^

In extended hosts such as CaWO_4_, Tb­(III) exhibits four equally spaced resonance lines separated by 252.5 G, attributed to hyperfine splitting from ^159^Tb (*I* = 3/2).^45^ Similar spin-Hamiltonian parameters have been reported for other solid-state hosts, including LiY_1–*x*_Tb_*x*_F_4_ which display clock transitions with energy gaps of ∼28 GHz.^46^ The tunnel splitting can vary widely with the coordination environment, ranging from 7.3 to 3.9 GHz in borate glasses^47^ to 5.40–6.90 GHz in crystalline lattices.^48^ Despite minor discrepancies in spectral assignment,^49^ these studies collectively demonstrate that Tb­(III) possesses an EPR active *pseudo*-doublet ground state with pronounced hyperfine structure and strong axial *g*-anisotropy. The following section presents the EPR characterization of our ∼*D*_6*h*_ Tb­(III) complex, extending these foundational observations to a molecular framework where the ligand-field symmetry and coordination environment can be precisely controlled.^50,51^

Guided by computational predictions (*vide supra*), we conducted a series of powder EPR measurements on the diluted ∼*D*_6*h*_ symmetric Tb­(III) complex **1@La**. As shown in Table 1, *ab initio* calculations suggest a low-lying transition with an energy gap of ∼0.8 GHz. The closest accessible experimental frequency is at L-band (∼1.1 GHz), however, no definitive EPR signal was observed under these conditions.

To investigate whether the absence of signal at L-band is caused by the underlying transition energies or limited due to the intrinsically low signal-to-noise (S/N) of L-band, we characterized **1@La** and a more diluted material **1′@La** via multifrequency EPR (Figure 4). The next highest commercially available microwave frequency is S-band at ca. 3.4 GHz: here we observe that both the more concentrated (**1@La** 6% Tb) and the more dilute (**1′@La** 0.7% Tb) samples exhibit a zero-field dominated spectrum (see Figure S16 a and c) similar to previously reported work on Tb­(III) in glasses.^52^ As our calculations propose a tunnel splitting of ∼1 GHz, and acknowledging that such calculations have historically underestimated the experimental values,^11,39^ the lowest accessible instrumental frequency was selected. Accordingly, S-band echo-detected field-swept (EDFS) measurements were attempted at 3.498 GHz pulse frequency (Figure S17a), the minimum operating frequency of the available spectrometer. Measurements were performed at 2.4 K using π/2 pulses of 16 ns. A shot repetition time (SRT) of 100 ns and 1000 shots per point (SPP) were used to enhance the S/N. Although features are observable in the resulting spectra (see Figure S17a), they do not correspond to the expected absorption profile obtained from the CW measurements, which could be due to the electron spin relaxation time in relation to the pulse timings used.

**Figure 4:**
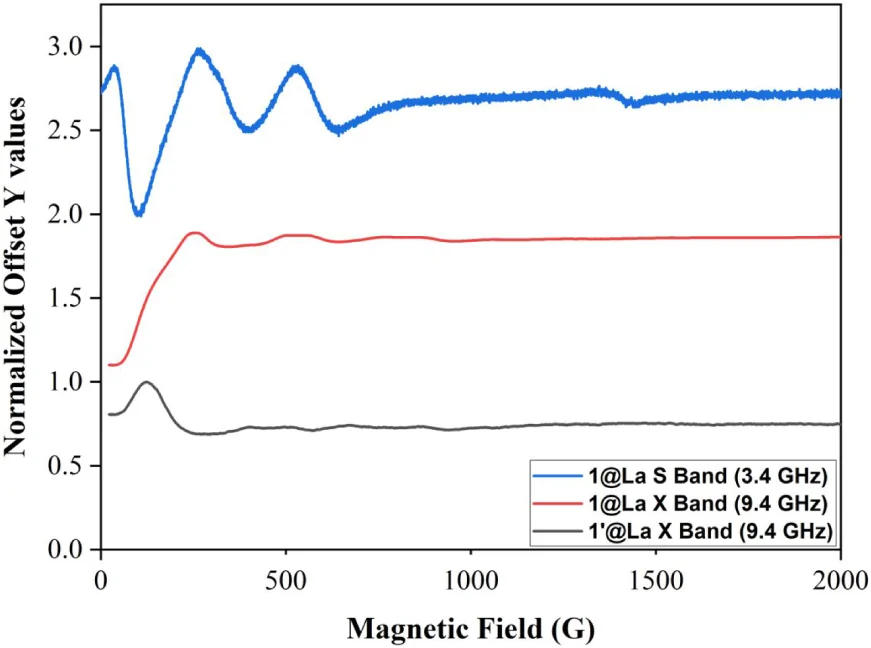
Continuous wave powder EPR spectra of **1@La** (S- and X-band) and **1′@La** (X-band) at 10 K.

The next step to further uncover the splitting at zero-field encountered is to continue increasing the microwave measurement frequency, which has the effect of pushing the same transition further upfield and uncovering more of the signal around zero-field. X-band (ca. 9.4 GHz), offering the most favorable S/N ratio among commercial EPR bands, further resolved this behavior. The measurement here uncovers a similar zero-field dominated spectrum of a strong negative (below the baseline) component along with three major features decreasing in intensity as the field is increased (Figure S16b and d). The extent in field of this spectrum is around 1000 G compared to around 800 G for the S-band spectrum. EDFS measurements were also attempted at X-band (Figure S17b). Further increasing the measurement frequency to K-band (Figure S18) and Q-band does not resolve any additional EPR signals from either of the Tb­(III) samples. The S/N obtained in the S- and X-band were satisfactory, indicating reliable instrumental performance under those experimental conditions. Accordingly, the measurements conducted at L-, K- and Q-band frequencies should not be fundamentally limited by the resonator response. Given the absence of any conclusive features at the higher frequency K- and Q-bands, we suggest that this observation is intrinsic to the energy levels of the samples, rather than being solely attributable to instrumental constraints.

Utilizing a variety of commercial X-band resonators, each with their own intrinsic dimensions, a different microwave frequency for measurement can be achieved for the same sample, analogous to a (hardware limited) frequency sweep (see Figure S19). Using this capability, a frequency difference of >0.3 GHz can be obtained. Measurements here show very similar spectra, though shifted, implying that the energy gap between the EPR active energy levels are almost invariant across a wide frequency range. This is further reinforced by the similarity to the S-band data (an almost 6 GHz difference) and could be considered favorable for future investigation of potential clock transition behavior.

Variable temperature investigations highlight the strong temperature dependence of the **1@La** resonance, with signals diminishing into the baseline above 30 K (Figure S16b) and lower (20 K) for the more dilute 0.7% Tb­(III) sample (Figure S16d). The more magnetically dilute 0.7% Tb sample begins to exhibit nonrandom orientations of the crystallites which can be seen from the narrower lines in the spectra. The signal intensity of the more dilute sample is comparable to the small Cu­(II) background signal present in the resonator at 3300 G (Figure S16d).

### EPR Analysis of **2**

An 8.8% Gd diluted analog of **2** was also prepared. This Gd­(III) sample, **2@La**, offers wider measurement opportunities arising from the *S* = 7/2 spin of Gd­(III) along with its differing relaxation profile, enabling measurement at higher temperature ranges. This approach has proven useful in the past as carried out by the Winpenny group.^18,53^ As expected, the differing relaxation profile of **2@La** compared to **1@La** enables measurements at much higher temperatures and across all available frequencies. Figure 5 displays the EPR spectra recorded at 10 K for each frequency band, providing a direct comparison of the signal characteristics across different microwave regions. The temperature dependence for each frequency, is provided in the Supporting Information (see Figure S20).

**Figure 5:**
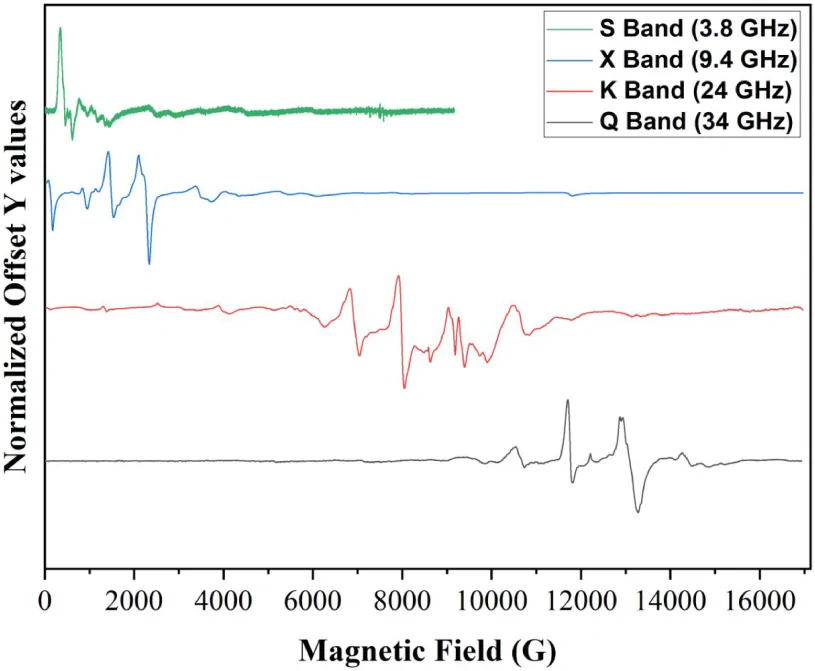
Multifrequency continuous wave EPR spectra of polycrystalline **2@La** as 8.8% Gd recorded at 10 K.

The spectra are dominated by axial zero-field splitting (ZFS), and a satisfactory simulation of the K-band spectrum at 293 K (Figure 6) can be achieved using only an isotropic *g*-value (1.999) and an axial ZFS parameter, *D* (0.136 cm^–1^). These parameters are almost identical to those obtained from fitting our temperature and field dependent magnetic data and are comparable to those reported by Winpenny’s group for ∼*D*_5*h*_ complexes.^22^ Good spectral agreement is found in the center of the spectrum with the fit diverging toward the wings of the spectrum, where field offsets become larger.

**Figure 6:**
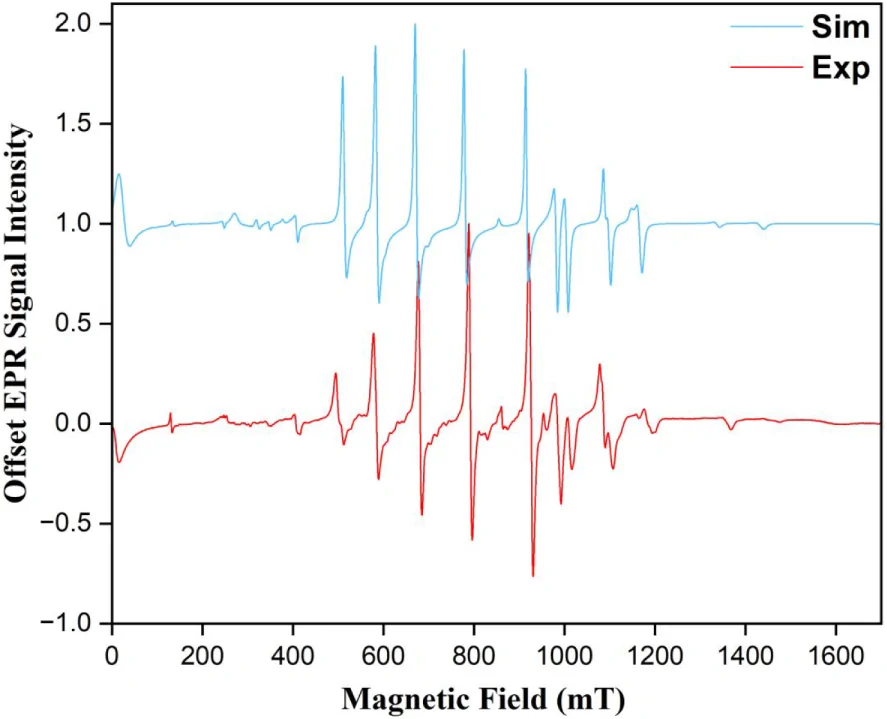
Experimental spectrum (red) at 293 K and simulation (blue) of polycrystalline **2@La** as 8.8% Gd at K-band, simulation parameters^40^ are S = 7/2, g = 1.999, *D* = 4084.9 MHz (0.136 cm^–1^), OptKnots = 60, lwpp = 1.4 mT.

As an orbital-quenched analog, the Gd­(III) complex provides a useful experimental assessment of the ligand-field symmetry relevant to the Tb­(III) system. Notably, the K-band spectrum can be reproduced very well using only the axial ZFS parameter *D* (equivalent to $3B_{2}^{0}$ ). The ability of this axial model to capture the number, relative intensities and line shapes of nearly all the features implies that the effects on the axial ligand field of, what might appear to be significant, distortions from *D*_6*h*_ symmetry are small. This observation is important in the context of the molecular design strategy and the requirements of a fine balance between axial purity and transverse mixing for opening a symmetry-protected tunneling gap in the Tb­(III) analog.

## Conclusions

We have studied a *pseudo*-*D*_6*h*_ Tb­(III) macrocyclic complex [Tb^III^(L^N6^)­(Ph_3_SiO)_2_]^+^ using *ab initio* calculations, magnetic and EPR measurements. Our *ab initio* calculations reveal the balance between the axial purity of the predominantly *m*_*J*_ = ±6 ground state and the transverse mixing in axially compressed *pseudo*-*D*_6*h*_ symmetry and we calculate a small tunneling gap ≈1 GHz. By calculating the tunneling gap for hexagonal vs nonhexagonal models, we find that $B_{6}^{\pm 6}{Ô}_{6}^{\pm 6}$ alone is not a sufficient transverse crystal field descriptor for the gap in **1**. This has important consequences for the design strategy and solely maximizing $B_{6}^{\pm 6}$ is too simplistic in *pseudo*-*D*_6*h*_ symmetry. The gap in **1** is not controlled by a single tunneling pathway, but emerges through competing contributions of the crystal-field Hamiltonian. Investigation of more ∼*D*_6*h*_ analogs e.g., with higher/lower distortion will allow the development of deeper insights into controlling the tunnel splitting of these Tb­(III)-based molecular systems. A more *D*_6*h*_-symmetric Tb­(III) analog could have either a smaller, similar or much larger gap, depending on the relative contributions of the surviving crystal field parameters and this represents an interesting synthetic target. Although our EPR results on La­(III)-diluted Tb­(III) powder samples are consistent with the possibility of a small tunneling gap, further work to improve signal-to-noise ratios, such as by growing larger single crystals, will be crucial for enhancing the clarity of the EPR spectra for further studies. It will also be interesting to examine the EPR spectra of [Tb^III^(L^N6^)­(Ph_3_SiO)_2_]^+^ in different crystal lattices, as the *ab initio* calculations suggest that the tunnel splitting is sensitive to the presence or absence of the counteranion. The Gd­(III) analog of the Tb­(III) complex provided wider measurement opportunities and the EPR spectra of [Gd^III^(L^N6^)­(Ph_3_SiO)_2_]^+^ are reproduced using only the axial ZFS parameter *D*, experimentally demonstrating that the ligand field remains largely axial in this *pseudo*-*D*_6*h*_ environment. The findings presented herein lay the groundwork for future studies that will advance our understanding of high-symmetry lanthanide complexes and their potential applications in quantum information processing.

## Supplementary Material



## Notes

Experimental methods, crystallographic data, computational results, and EPR data (PDF)
