# Supplementary material for: Magnetic, EPR, and Computational Insights into ∼D 6h Tb(III) and Gd(III) Complexes
Source: Inorg Chem. 2026 Aug 7;65(33):19118–26. doi: 10.1021/acs.inorgchem.6c01628 (PMC13509004; doi:10.1021/acs.inorgchem.6c01628)
Supplement: Supplementary file 1 [file ic6c01628_si_001.pdf]

Supporting Information

Magnetic, EPR and Computational Insights into ~D_{6h} Tb(III) and Gd(III) Complexes

Jishnu Gangadharan,[1] Adam Brookfield,[2] Tanu Sharma,[3] Ethan Lowe,[1] Claire Wilson,[1] Angelos B. Canaj,[1] Gopalan Rajaraman,*[3] David Collison*[2] and Mark Murrie*[1]

1. School of Chemistry, University of Glasgow, University Avenue, Glasgow G12 8QQ, U.K. E-mail: mark.murrie@glasgow.ac.uk

2. Department of Chemistry, The University of Manchester, Oxford Road, Manchester M13 9PL, U.K. E-mail: david.collison@manchester.ac.uk

3. Department of Chemistry, Indian Institute of Technology Bombay, Powai, Mumbai, Maharashtra 400076, India. E-mail: rajaraman@chem.iitb.ac.in

Table of Contents

1. Experimental Methods S2

2. Synthesis and Characterization S3

3. Theoretical Calculations S14

4. EPR Analysis S22

5. References S27

Experimental Methods

All experiments were carried out under aerobic conditions using materials and solvents as received without any further purification.

Single crystal X-ray diffraction data for **1** were collected at the ESPRC National Crystallographic Service at the University of Southampton. Data for **2** and **3** were collected on a Bruker D8 Venture goniometer with a Bruker PHOTON II CPAD detector and dual IμS 3.0 microfocus sources (Cu and Mo Kα) equipped with an Oxford Cryosystems Cryostream 1000 device at the University of Glasgow (full crystallographic details are provided in the CIFs deposited with CCDC, numbers provided in Table S1). Powder X-ray diffraction patterns were collected on freshly prepared samples of **1**, **2** and **3** on a Rigaku MiniFlex benchtop diffractometer equipped with a Cu sealed tube X-ray source (λ (Cu-Kα) = 1.5405 Å) and a six-position sample changer on zero-background silicon sample holders at the University of Glasgow. Elemental analyses (C, H, N) for compound **1** were carried out using an Exeter CE-440 analyzer by the University of Glasgow's microanalysis service, while compounds **2** and **3** were analyzed with a Thermo FlashEA 1112 CHNS-O Elemental Analyzer operated by MEDAC Ltd. IR spectra of complexes **1**, **2** and **3** were measured using a JASCO FTIR-4100 spectrophotometer.

EDX-SEM measurements were performed at the University of Glasgow on the diluted samples **1@La, 1'@La** and **2@La** using a TESCAN CLARA Ultra High-Resolution Scanning Electron Microscope with a Field Emission Gun electron source and an Oxford Instruments UltimMax 65 with Aztec live interface with a Schottky field electron emitter with a voltage range of 0.5 – 30 keV and probe current range of 2 – 400 nA.

Magnetic measurements for **1** and **2** were carried out using a Quantum Design MPMS3 EverCool SQUID magnetometer equipped with a 7 T magnet at the University of Glasgow. The crystalline samples were ground to a powder, restrained in eicosane and placed in gelatine capsules in straws. The data were corrected for the diamagnetic contributions from the sample holder and eicosane (blank measurements) and for the diamagnetism of all ions using Pascal's constants.

Continuous wave (CW) EPR spectra were obtained at microwave frequencies, L-band (*ca.* 1.1 GHz), S-band (*ca.* 3.4 GHz), X-band (*ca.* 9.4 GHz), K-band (*ca.* 24 GHz) and Q-band (*ca.* 34 GHz) across the Bruker EMX and Bruker E580X spectrometer platforms. X-band data were measured using a Bruker 4122-SHQ resonator or Bruker Flexline 4118X-MD5. The other frequencies were measured using a commercial Bruker Flexline resonator specific to each frequency. Namely, the 4118L-MS5 & 4118S-MS5 for L- and S-band along with the ER6706-KT & ER5106-QT for K-band and Q-band. Cryogenic temperatures were achieved using two closed cycle He cryocoolers, the Cryogenic Ltc. CF-VTC (for < 5 K) or a ColdEdge Stinger mated to an Oxford Instruments ESR900 or CF935 (depending on the type of resonator in use). The dried crystals were packed into a 3 mm OD (2 mm ID) quartz EPR tube (QSIL, DE) to maximize resonator fill height and to fit across as many resonators as possible without exchange of tube, except in the case of Q-band, where a 2 mm OD (1 mm ID) tube must be

used owing to resonator dimensions. Typical measurement parameters used were: modulation amplitude 2-5 G, Microwave power 2.3-1.2 mW. Power was varied to account for the differing relaxation profiles between the Tb and Gd doped samples and maintain non-saturating measurement conditions. Reported fields were corrected against a Bruker Strong Pitch standard sample (g = 2.0028) and scaled to the microwave frequency values in the legend of each plot.

Synthesis and characterization

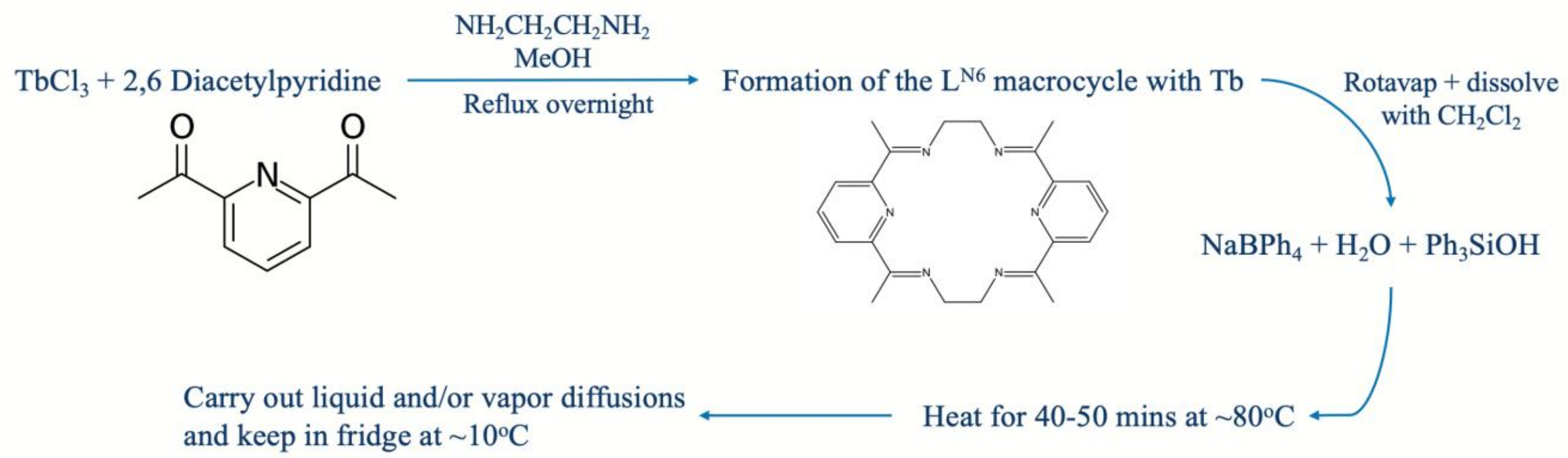

Scheme S1: Schematic flowchart representing the synthetic strategy used to produce **1**. $GdCl_3{\cdot}6H_2O$ is used instead of $TbCl_3{\cdot}6H_2O$ to produce **2**.

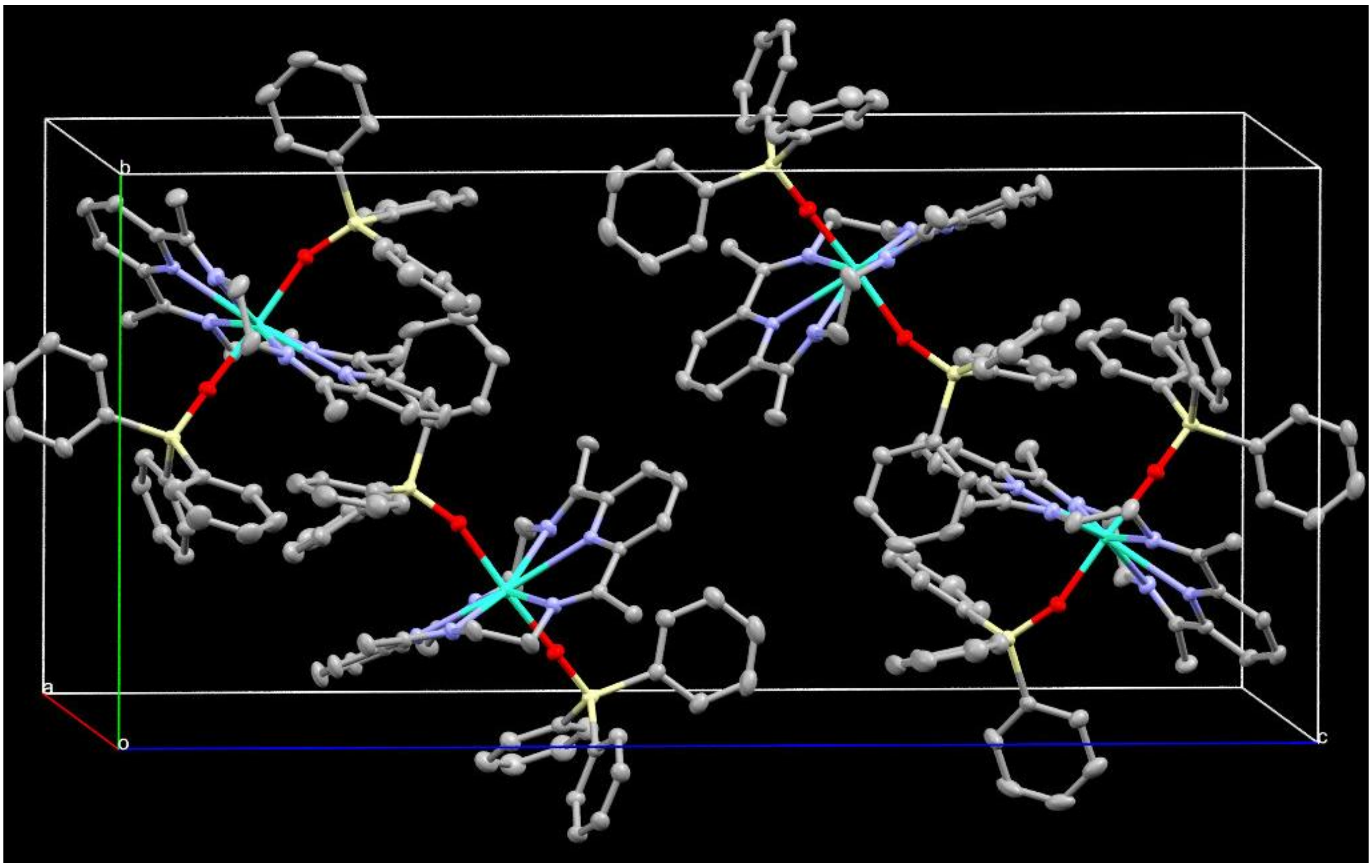

Figure S1. Crystal packing of **1** highlighting the packing between neighboring complexes. H-atoms and counterions omitted for clarity.

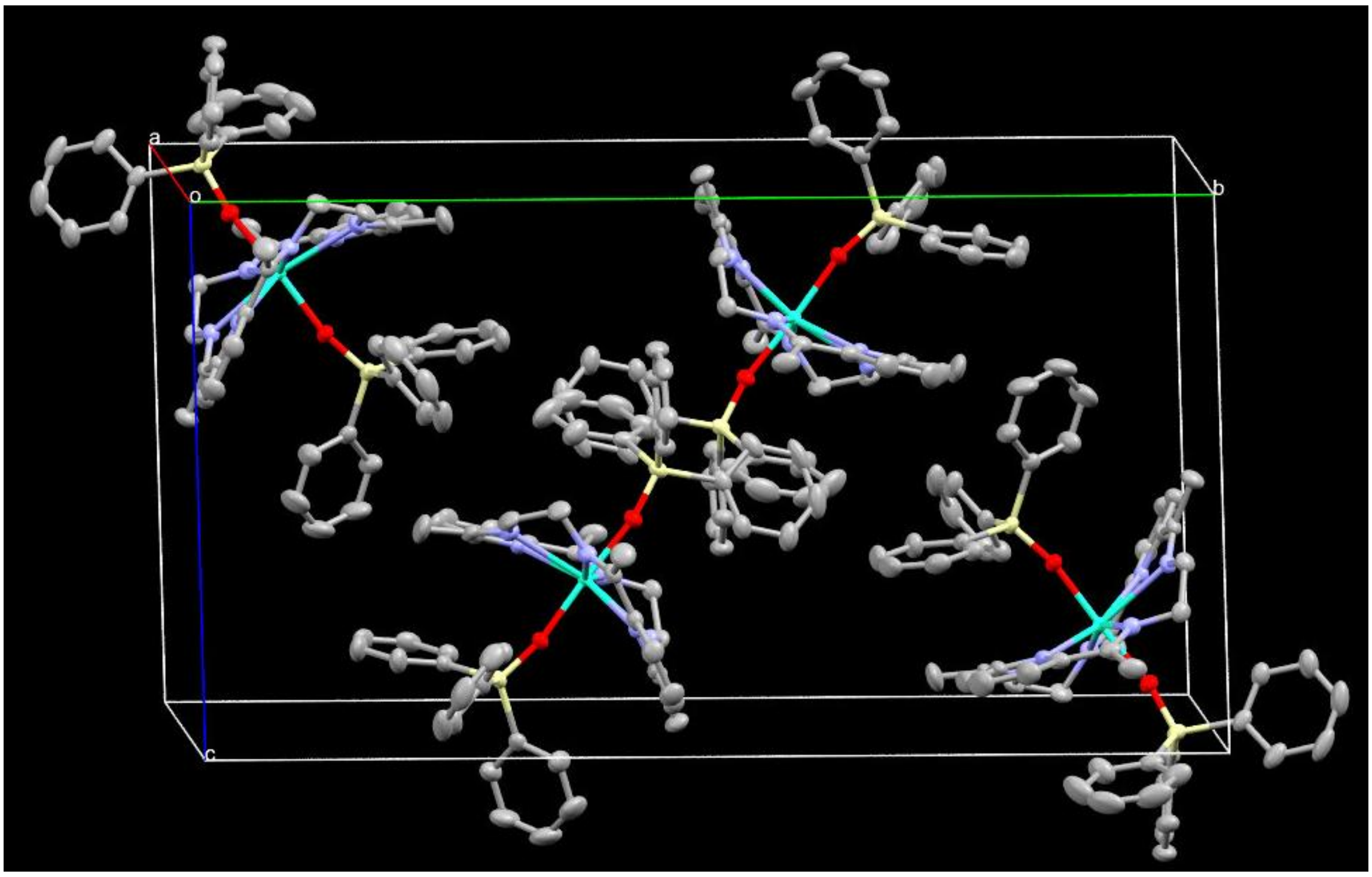

Figure S2. Crystal packing of the previously published Dy(III) analog highlighting the packing between neighboring complexes. H-atoms and counterions omitted for clarity.[1]

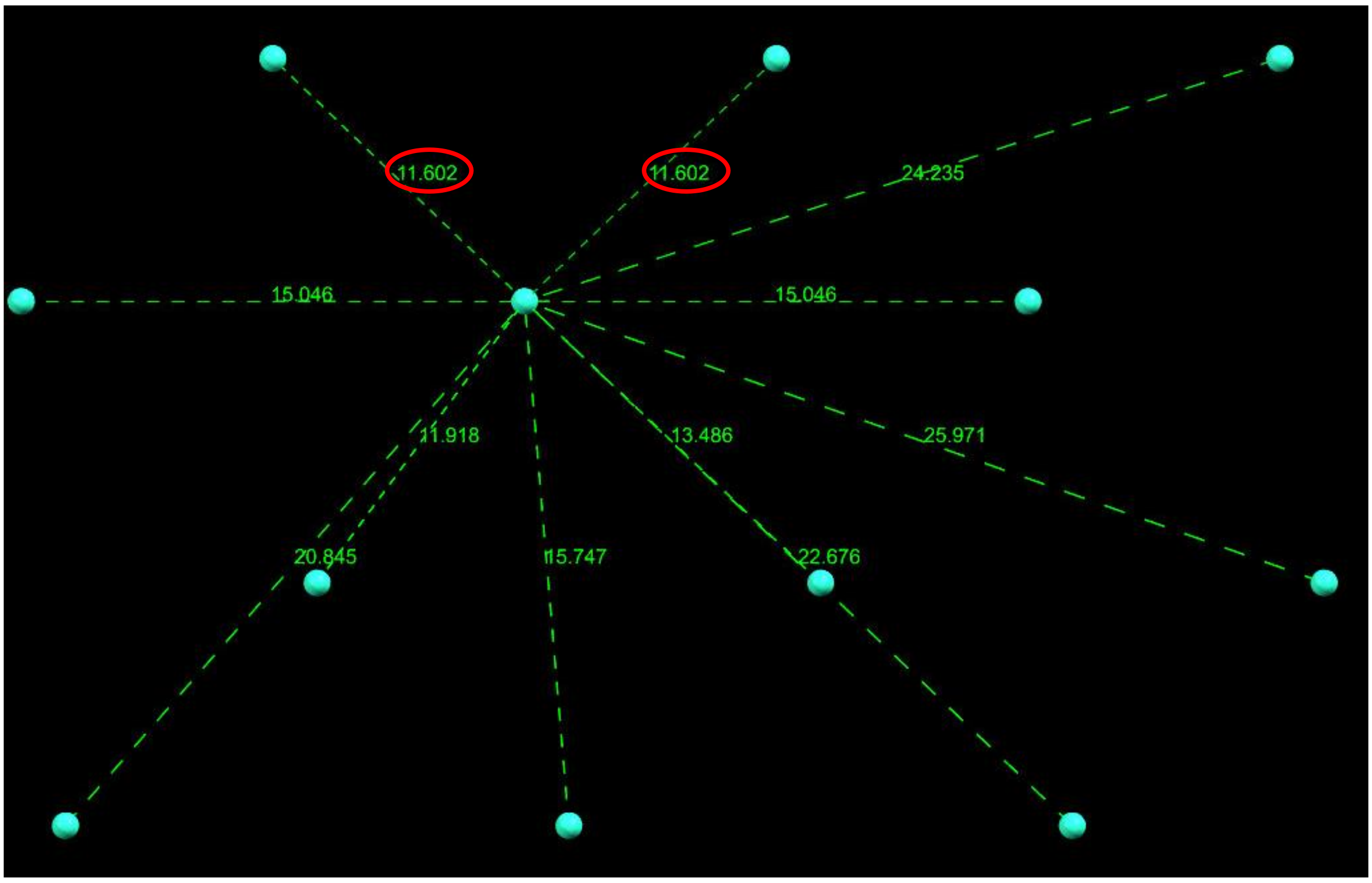

Figure S3. Diagram illustrating a 3 x 3 array of Tb(III) centers, with the shortest intermolecular Tb···Tb separation highlighted in red.

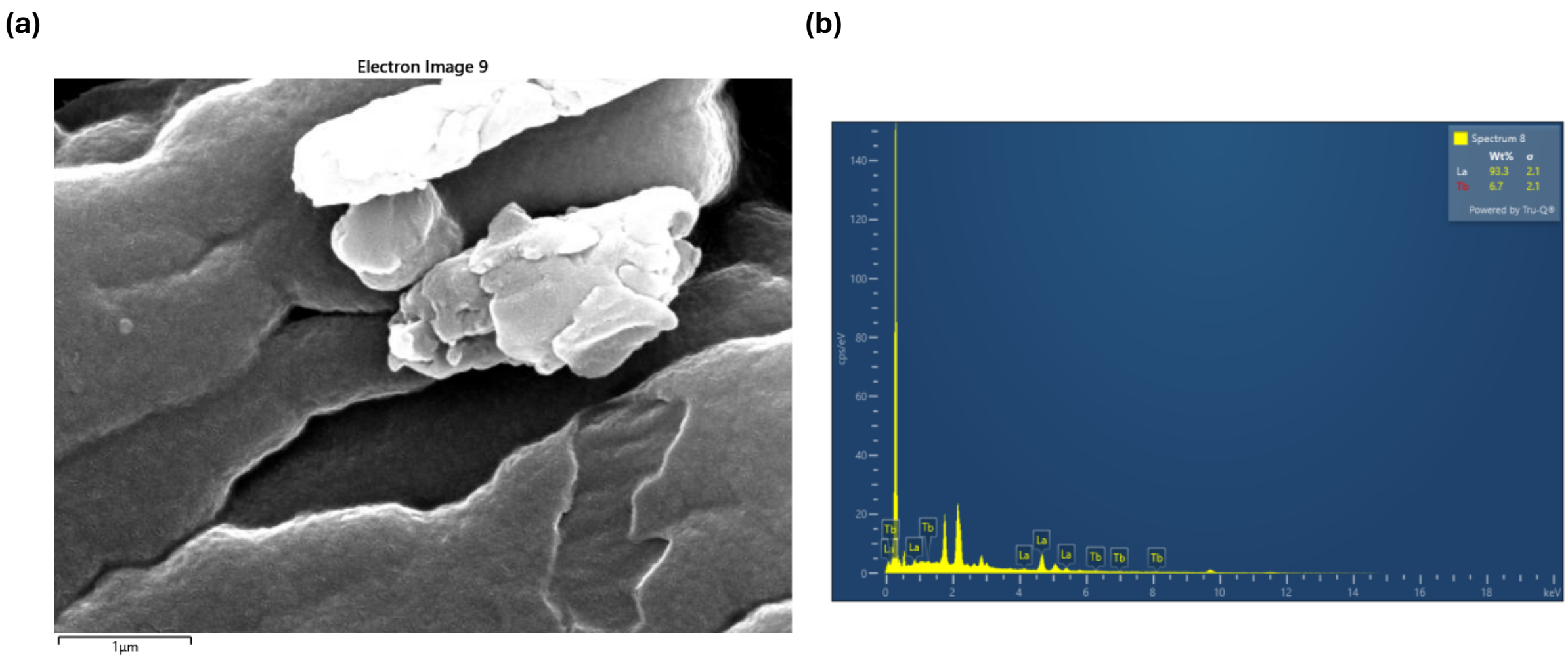

Figure S4. (a) High resolution SEM image captured at 25,000x magnification of **1@La** doped 6 % Tb and (b) EDX report of the as synthesized sample.

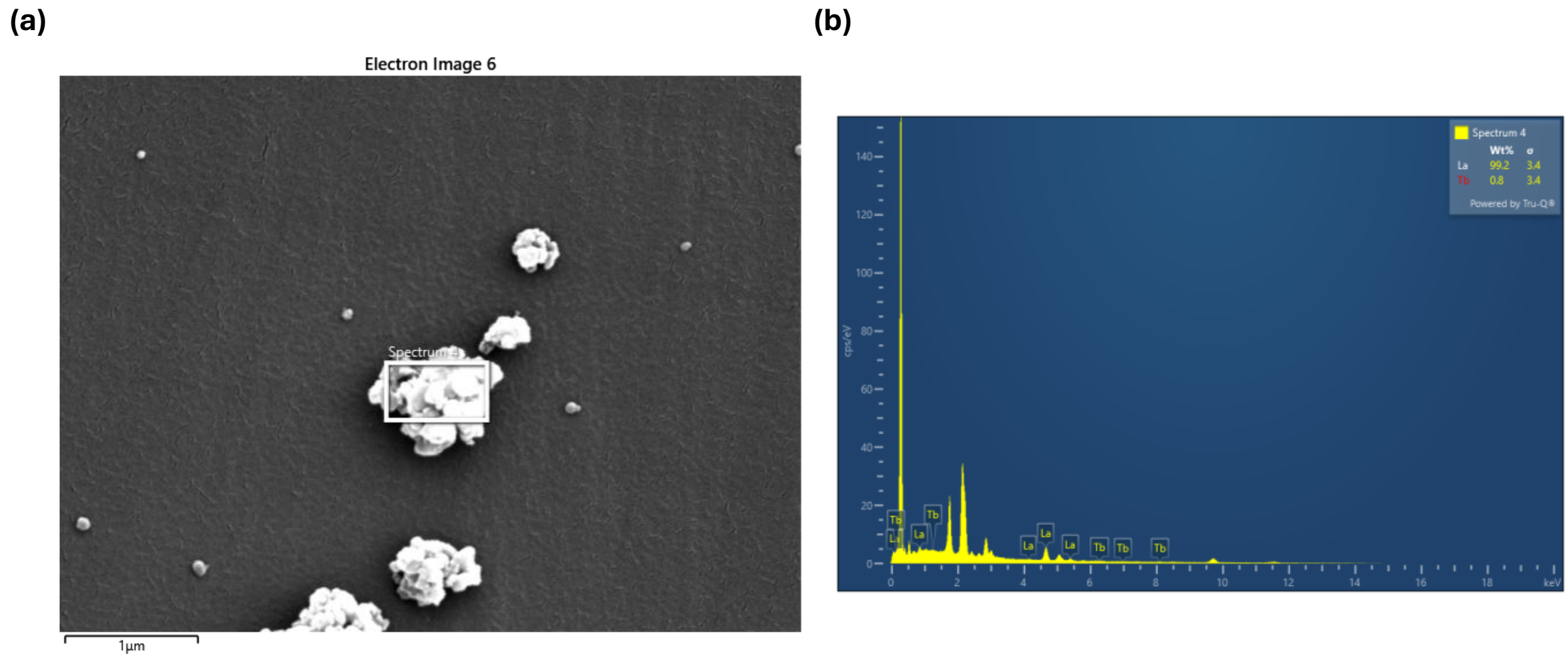

Figure S5. (a) High resolution SEM image captured at 25,000x magnification of **1'@La** doped 0.7% Tb and (b) EDX report of the as synthesized sample.

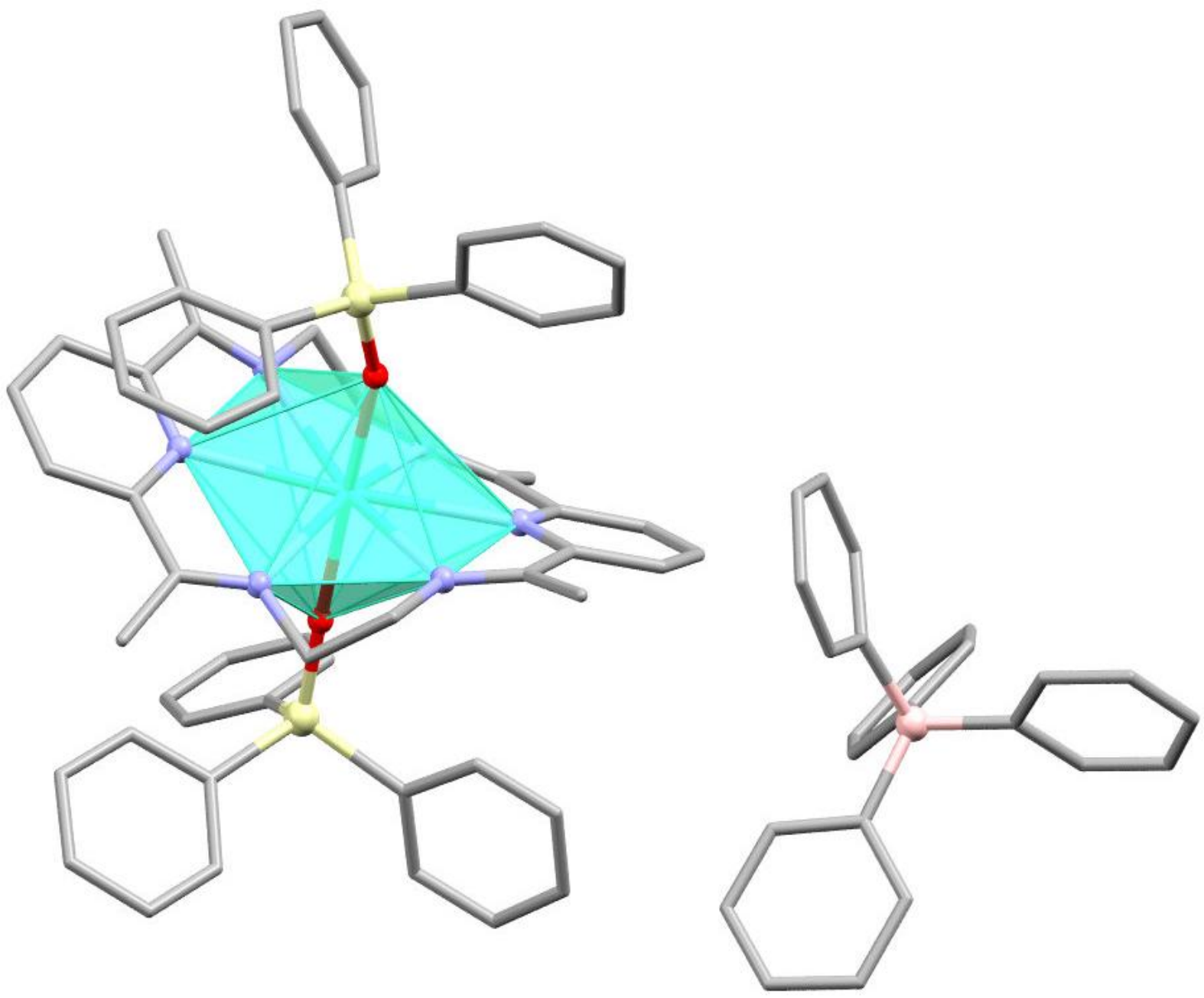

Figure S6. Structure of **2**. Gd (cyan); C (grey); N (blue); O (red); Si (yellow); B (pink); H omitted for clarity.

(a) **(b)**

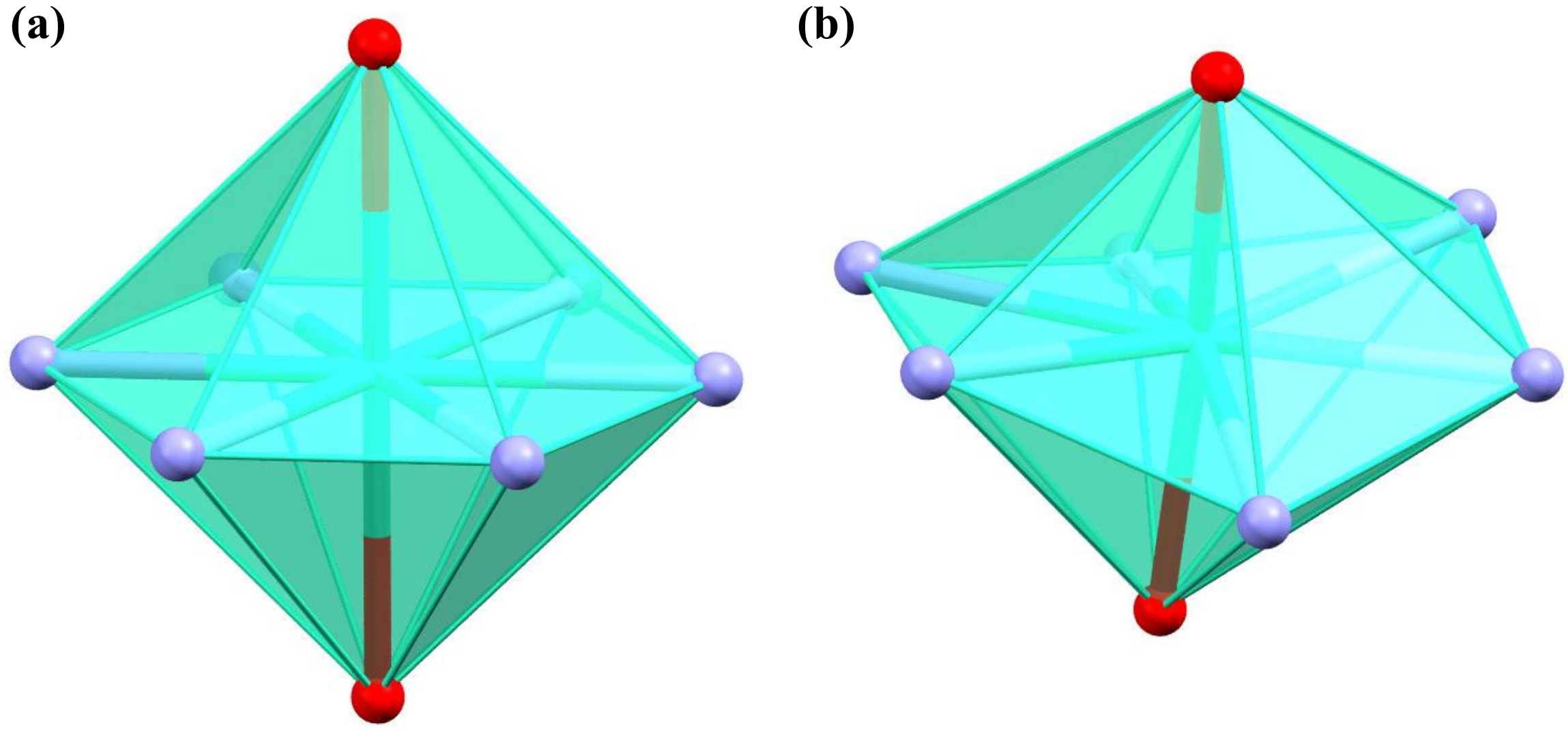

Figure S7. Representation of (a) the ideal hexagonal bipyramidal structure with (b) the structure of **1** with the first coordination sphere to show the distortion of its geometry.

(a) **(b)**

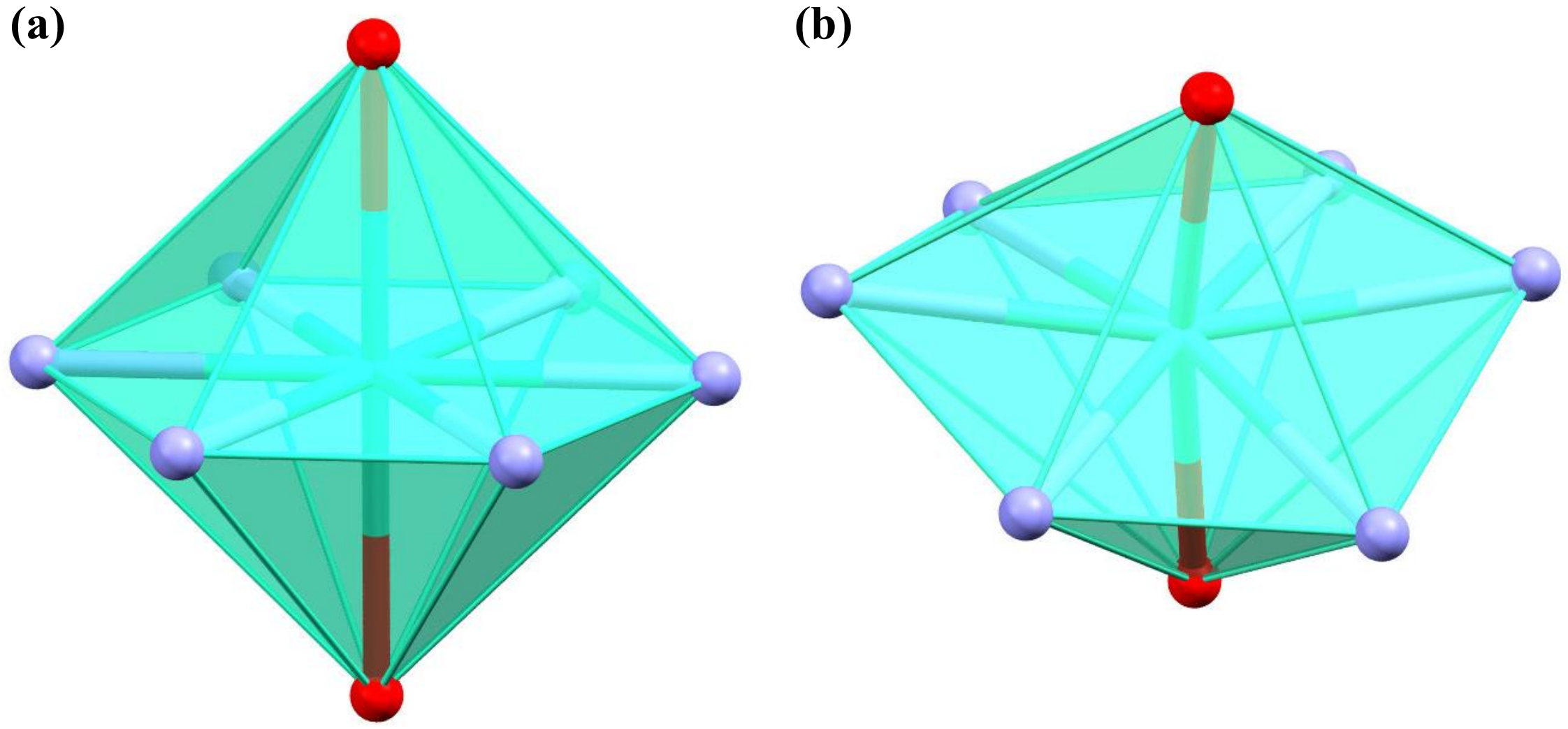

Figure S8. Representation of (a) the ideal hexagonal bipyramidal structure with (b) the structure of **2** with the first coordination sphere to show the distortion of its geometry.

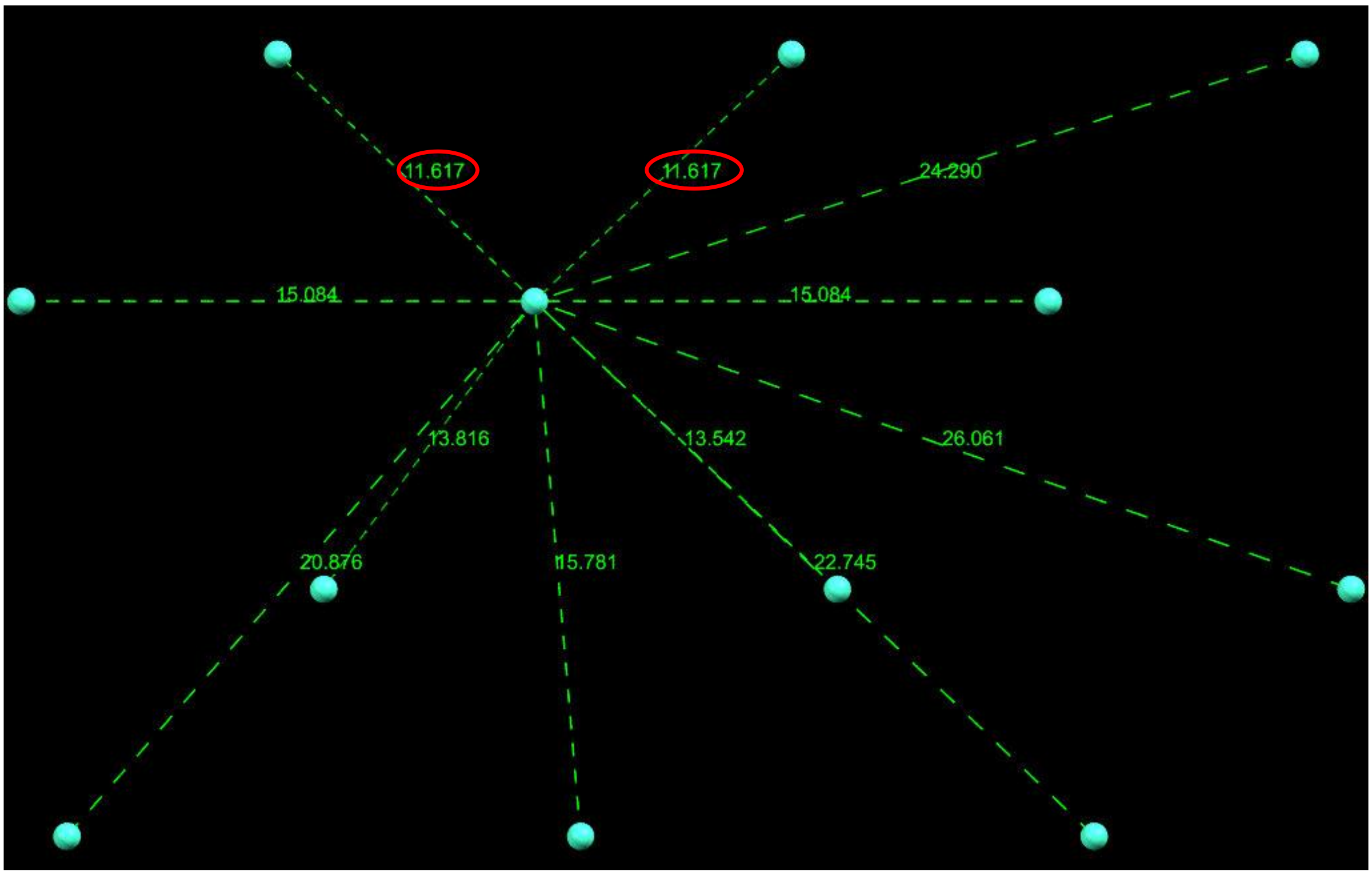

Figure S9. Diagram illustrating a 3 x 3 array of Gd(III) centers, with the shortest intermolecular Gd···Gd separation highlighted in red.

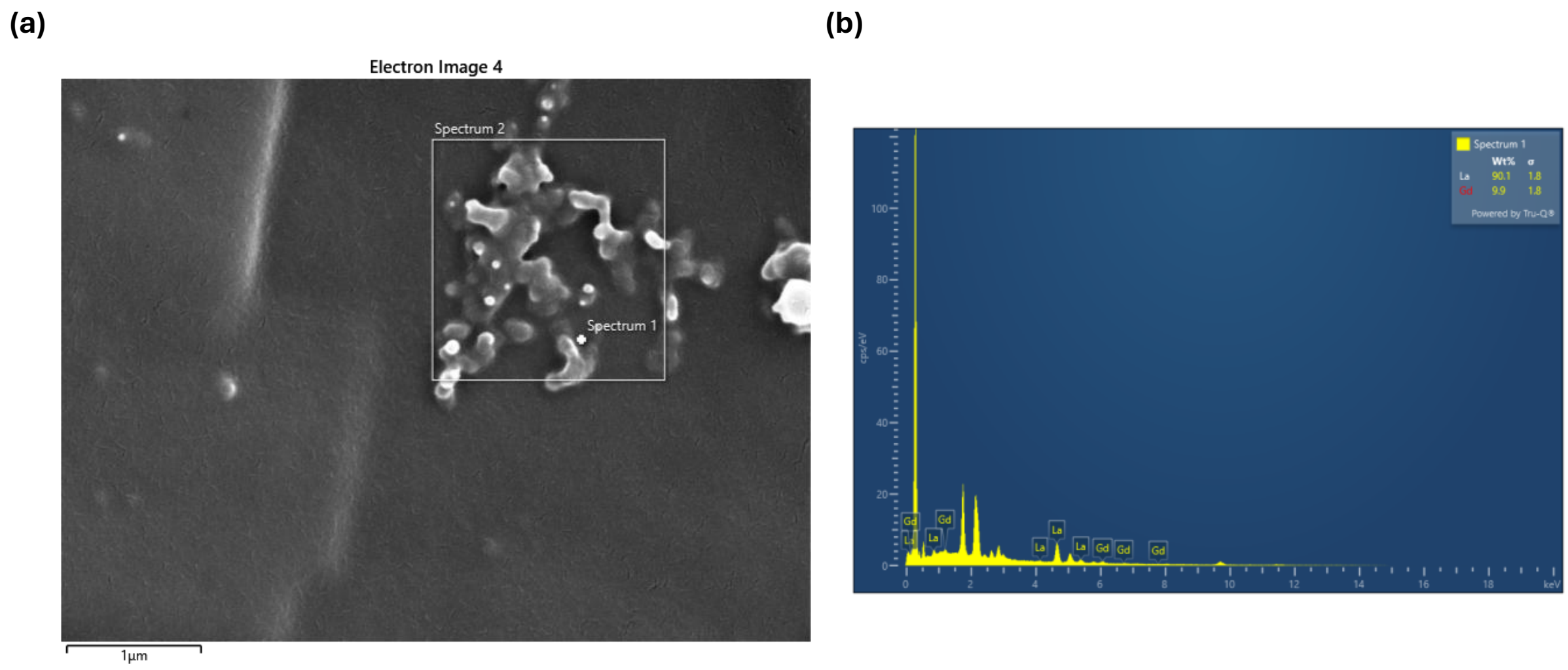

Figure S10. (a) High resolution SEM image captured at 25,000x magnification of **2@La** doped 8.8% Gd and (b) EDX report of the as synthesized sample for spectrum 1 (area) on the SEM image respectively.

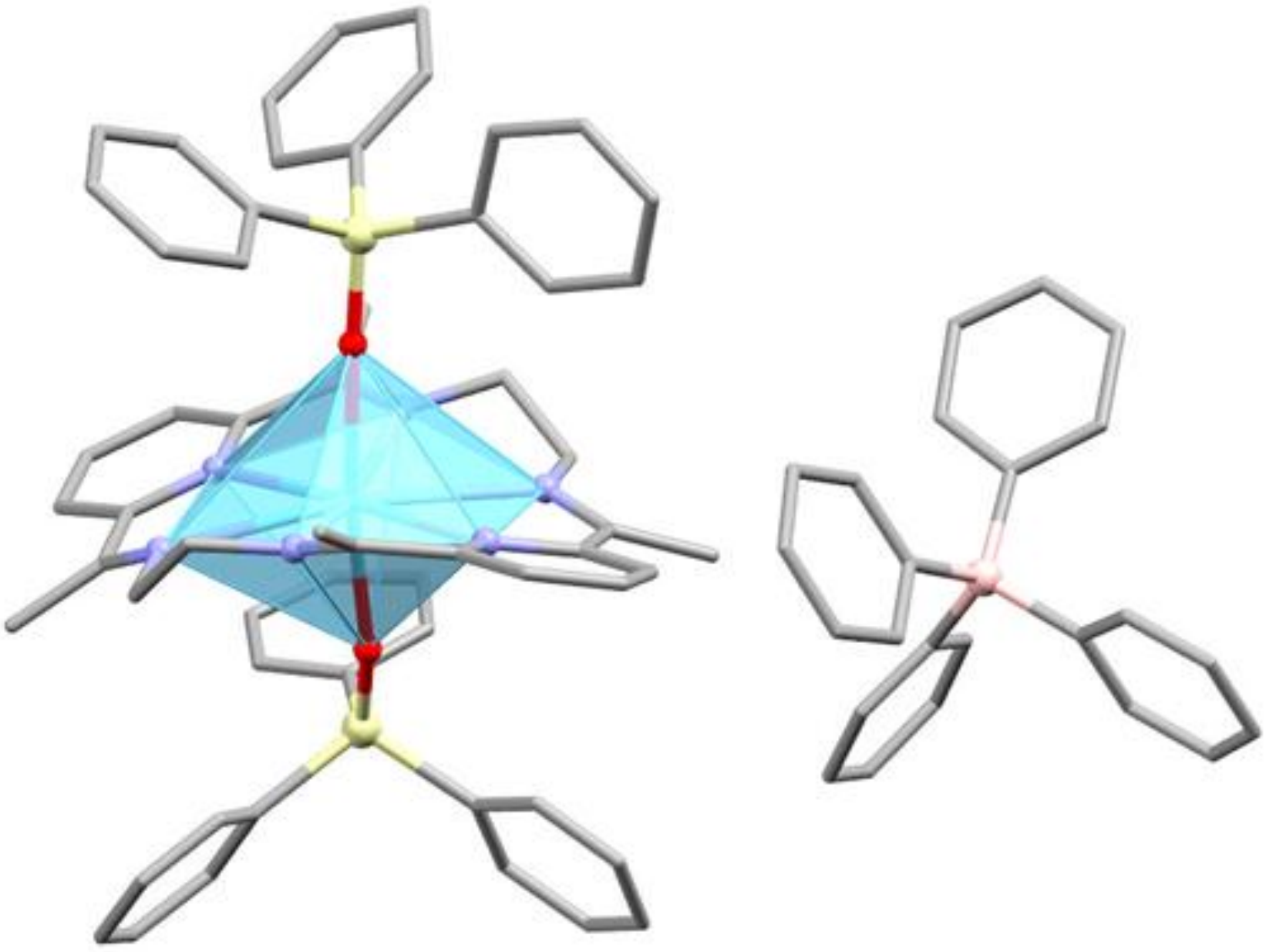

Figure S11. Structure of **3**. La (blue); C (grey); N (blue); O (red); Si (yellow); B (pink); H omitted for clarity.

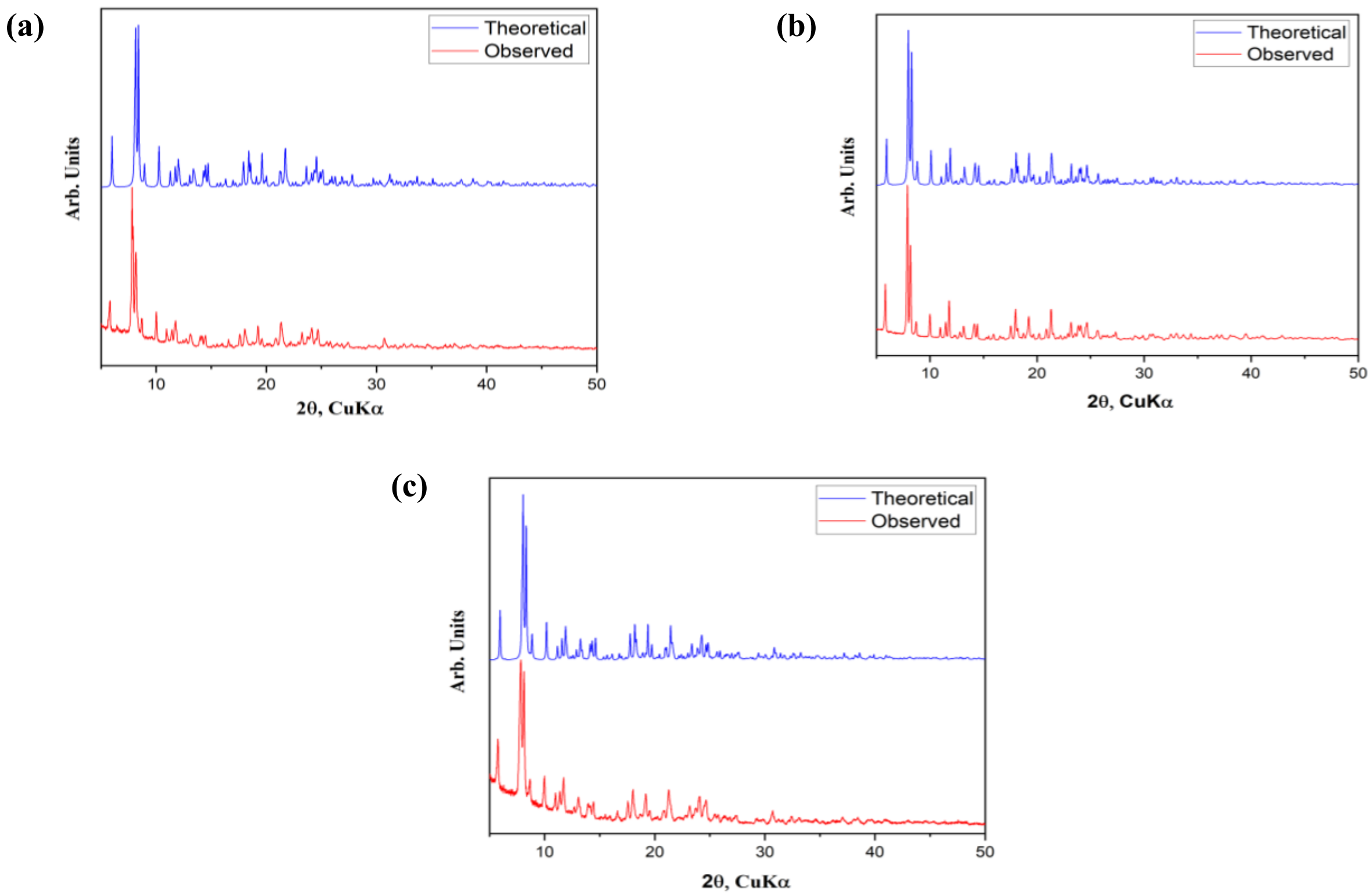

Figure S12. Calculated and observed PXRD patterns of isostructural complexes (a) $[Tb^{III}L^{N6}(Ph_3SiO)_2]BPh_4$ (**1**); (b) $[La^{III}L^{N6}(Ph_3SiO)_2]BPh_4$ (**3**) and (c) $[Gd^{III}L^{N6}(Ph_3SiO)_2]BPh_4$ (**2**), which crystallize in the $P2_1/c$ space group.

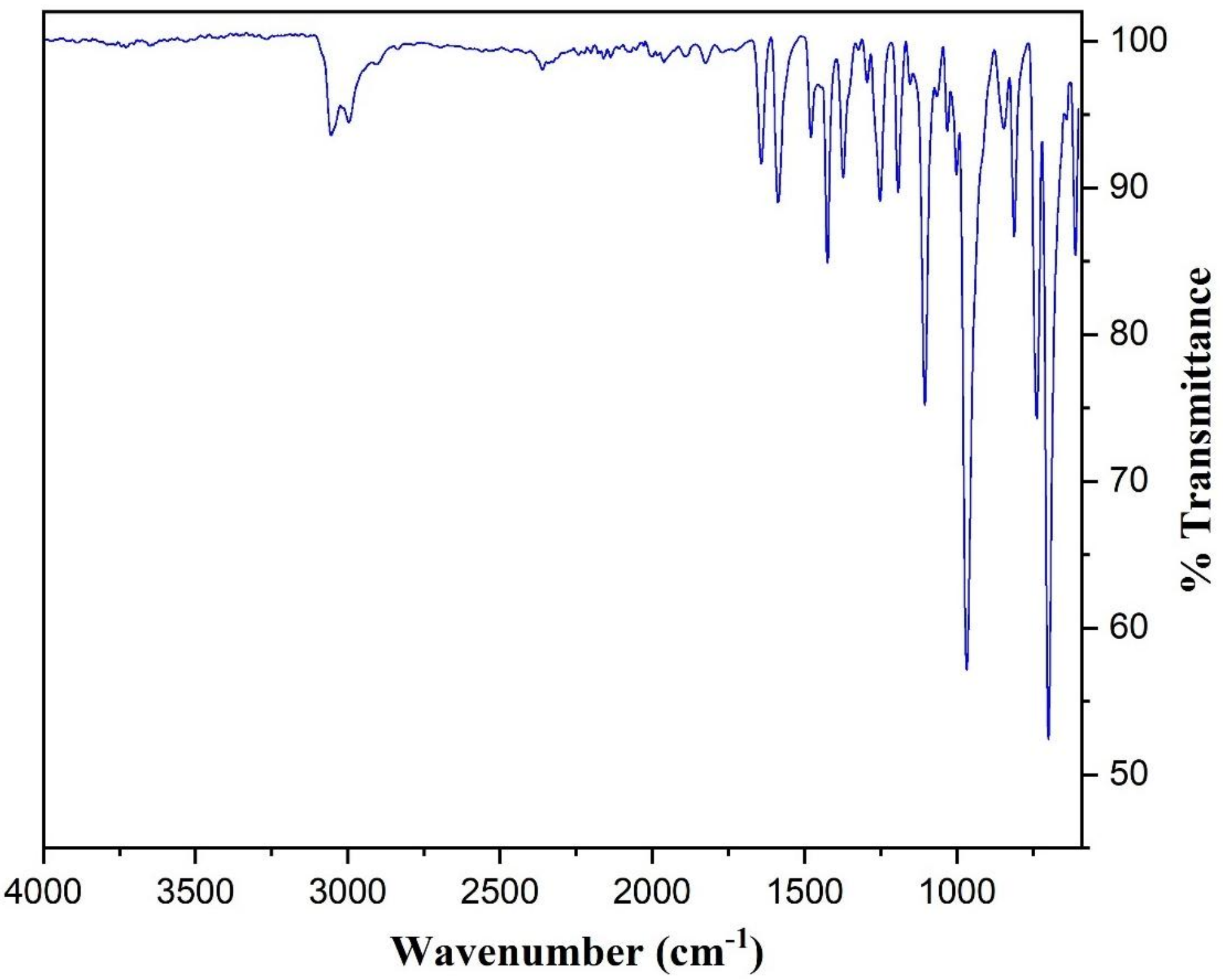

Figure S13. Infrared spectrum of compound **1**.

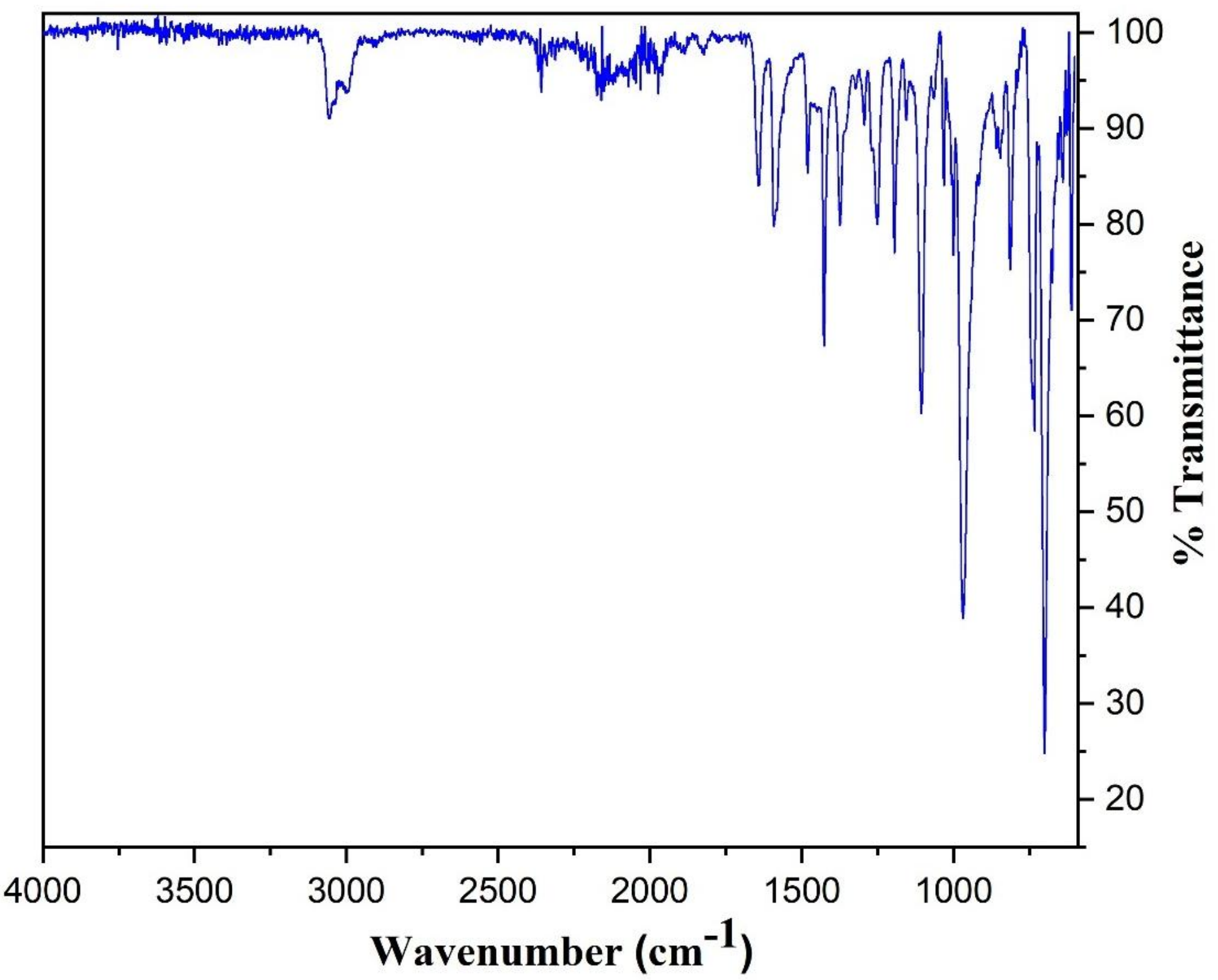

Figure S14. Infrared spectrum of compound **2**.

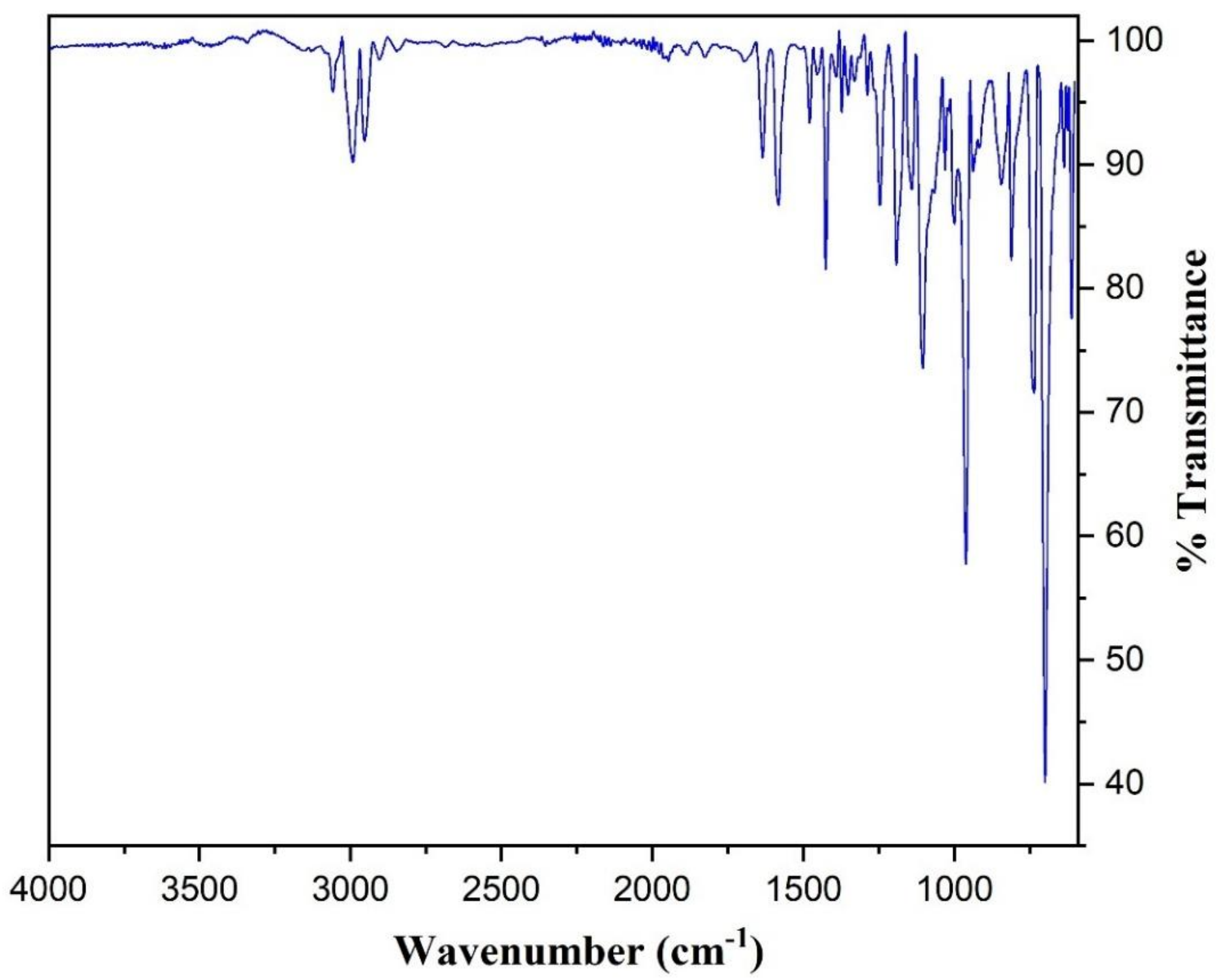

Figure S15. Infrared spectrum of compound **3**.

Table S1. Crystal Data and Structure Refinement Parameters for **1**, **2** and **3**.

	**1**	**2**	**3**
Empirical formula	$C_{82}H_{76}BN_6O_2Si_2Tb$	$C_{82}H_{76}BGdN_6O_2Si_2$	$C_{82}H_{76}BLaN_6O_2Si_2$
**CCDC number**	2536973	2536974	2536975
Formula weight	1403.39	1401.72	1383.38
Temperature/K	100.00	100.0	298
Crystal system	monoclinic	monoclinic	monoclinic
Space group	$P2_1/c$	$P2_1/c$	$P2_1/c$
a/Å	14.7467(1)	14.7422(16)	14.8850(4)
b/Å	15.0457(1)	15.0839(14)	15.3298(4)
c/Å	31.3829(3)	31.446(4)	32.0162(10)
α/°	90	90	90
β/°	92.519(1)	92.530(4)	92.512(1)
γ/°	90	90	90
Volume/$Å^3$	6956.33(9)	6985.8(13)	7298.6(4)
Z	4	4	4
$\rho_{calc}g/cm^3$	1.340	1.333	1.259
μ/mm^{-1}	1.10	1.04	0.67
F(000)	2896.0	2892.0	2864.0
Crystal size/mm^3	0.18 × 0.08 × 0.04	0.25 × 0.09 × 0.07	0.8 × 0.24 × 0.16
Radiation (Å)	MoKα (λ = 0.71073)		
2Θ range for data collection/°	4.0 to 76.3	2.6 to 56.6	4.5 to 54.2
Index ranges	$-25 \le h \le 25$, $-25 \le k \le 26$, $-53 \le l \le 53$	$-19 \le h \le 17$, $-20 \le k \le 20$, $-41 \le l \le 41$	$-16 \le h \le 19$, $-19 \le k \le 19$, $-41 \le l \le 41$
Reflections collected	348524	128557	81272
Independent reflections	36675 [R_{int} = 0.0365, R_{sigma} = 0.0206]	17308 [R_{int} = 0.0809, R_{sigma} = 0.0507]	16083 [R_{int} = 0.0479, R_{sigma} = 0.0343]
Data/restraints/parameters	36675/0/851	17308/0/851	16083/420/851
Goodness-of-fit on F^2	1.063	1.031	1.014
Final R indexes [I>=2σ (I)]	R_1 = 0.029, wR_2 = 0.069	R_1 = 0.038, wR_2 = 0.079	R_1 = 0.0462, wR_2 = 0.1222
Final R indexes [all data]	R_1 = 0.036, wR_2 = 0.069	R_1 = 0.058, wR_2 = 0.086	R_1 = 0.0630, wR_2 = 0.1360
Largest diff. peak/hole / e $Å^{-3}$	2.09/−0.82	1.16/−0.48	1.11/−0.85

Table S2. SHAPE analysis results for **1**, **2** and **3**. Bold font highlights the lowest value, which corresponds to the least distorted symmetry.

**Molecular geometry shape**	**1 (Tb)**	**2 (Gd)**	**3 (La)**
Octagon	30.382	30.360	29.964
Heptagonal pyramid	21.264	20.861	20.485
**Hexagonal bipyramid**	**2.029**	**1.978**	**1.433**
Cube	10.508	10.448	9.888
Square antiprism	16.153	15.884	16.356
Triangular dodecahedron	14.023	13.873	14.188
Johnson gyrobifastigium J26	6.193	6.187	7.212
Johnson elongated triangular bipyramid J14	21.293	21.243	21.402
Biaugmented trigonal prism J50	14.849	14.679	15.356
Biaugmented trigonal prism	14.680	14.495	15.119
Snub diphenoid J84	14.567	14.396	15.237
Triakis tetrahedron	11.341	11.280	10.718
Elongated trigonal bipyramid	18.645	18.480	18.555

Table S3. Selected bond lengths for **1**, **2** and **3**.

Atom	Atom	Length (Å)		
		**1 (Tb)**	**2 (Gd)**	**3 (La)**
Ln	O1	2.1497(8)	2.1902(18)	2.277(3)
Ln	O2	2.1659(8)	2.1750(18)	2.268(3)
Ln	N1	2.6555(9)	2.678(2)	2.735(3)
Ln	N2	2.6452(9)	2.648(2)	2.719(3)
Ln	N3	2.6510(9)	2.626(2)	2.713(3)
Ln	N4	2.6733(9)	2.662(2)	2.729(3)
Ln	N5	2.6411(10)	2.648(2)	2.698(3)
Ln	N6	2.6202(10)	2.653(2)	2.709(3)

Table S4. Selected bond angles for **1**, **2** and **3**.

Atom	Atom	Atom	Angle/°		
			**1 (Tb)**	**2 (Gd)**	**3 (La)**
O1	Ln	O2	170.74(3)	170.69(7)	170.52(11)
N1	Ln	N2	60.12(3)	59.63(7)	58.94(8)
N1	Ln	N6	60.25(3)	59.52(7)	59.13(9)
N2	Ln	N3	62.60(3)	63.61(7)	62.97(9)
N4	Ln	N3	59.58(3)	60.20(7)	59.29(9)
N5	Ln	N4	59.72(3)	59.99(7)	59.27(9)
N5	Ln	N6	63.60(3)	62.78(7)	63.69(10)

Theoretical Calculations

Table S5. Energies, tunnel splitting, *g*-tensors and the angle between the ground state g_{zz} to the excited state g_{zz} in **1** and $\mathbf{1}_{wc}$ ($\mathbf{1}_{wc}$ being **1** without the counterion).

**Energy states**	**Energies (cm^{-1})**	**Tunnel Splitting (cm^{-1})**	g_{zz}	**Angle (°) with ground g_{zz}**
		**1**		
1	0.000 / 0.028	2.80×10^{-2}	17.9	-
2	113.5 / 113.7	1.67×10^{-1}	15.3	19.8
3	283.3 / 284.7	1.36×10^{0}	11.7	19.7
4	491.1 / 567.1	7.61×10^{1}	8.30	18.8
5	794.4 / 820.2	2.58×10^{1}	5.10	19.2
6	1046 / 1071	2.42×10^{1}	2.20	20.2
7	1173	-	-	-
		$\mathbf{1}_{wc}$		
1	0.000 / 0.031	3.10×10^{-2}	17.9	-
2	119.7 / 119.9	1.51×10^{-1}	15.3	18.9
3	295.3 / 296.6	1.34×10^{0}	11.7	18.8
4	506.4 / 583.9	7.75×10^{1}	8.30	18.5
5	814.9 / 838.8	2.39×10^{1}	5.10	18.8
6	1070 / 1091	2.10×10^{1}	2.30	20.2
7	1197	-	-	-

Table S6. Composition of energy states in **1** (full numerical precision shown for all states; 6 doublets and 1 singlet state respectively).

Energy state	Composition of the states
1	0.497335 |+6⟩ + 0.497335 |−6⟩<br>0.000005 |+5⟩ + 0.000005 |−5⟩<br>0.002358 |+4⟩ + 0.002358 |−4⟩<br>0.000189 |+3⟩ + 0.000189|−3⟩<br>0.000006 |+2⟩ + 0.000006 |−2⟩<br>0.000058 |+1⟩ + 0.000058 |−1⟩<br>0.000103 |0⟩
	0.497348 |+6⟩ + 0.497348 |−6⟩<br>0.000006 |+5⟩ + 0.000006 |−5⟩<br>0.002381 |+4⟩ + 0.002381 |−4⟩<br>0.000196 |+3⟩ + 0.000196 |−3⟩<br>0.000027 |+2⟩ + 0.000027 |−2⟩<br>0.000038 |+1⟩ + 0.000038 |−1⟩<br>0.000001 |0⟩
2	0.00032 |+6⟩ + 0.00032 |−6⟩<br>0.45240 |+5⟩ + 0.45240 |−5⟩<br>0.04593 |+4⟩ + 0.04593 |−4⟩<br>0.00091 |+3⟩ + 0.00091 |−3⟩<br>0.00007 |+2⟩ + 0.00007 |−2⟩<br>0.00036 |+1⟩ + 0.00036 |−1⟩<br>0.00003 |0⟩
	0.000309 |+6⟩ + 0.000309 |−6⟩<br>0.452528 |+5⟩ + 0.452528 |−5⟩<br>0.046013 |+4⟩ + 0.046013 |−4⟩<br>0.000822 |+3⟩ + 0.000822 |−4⟩<br>0.000060 |+2⟩ + 0.000060 |−2⟩<br>0.000035 |+1⟩ + 0.000035 |−1⟩

	0.000473 $\|0\rangle$
	0.00211 $\|+6\rangle$ + 0.00211 $\|-6\rangle$<br>0.04045 $\|+5\rangle$ + 0.04045 $\|-5\rangle$<br>0.34346 $\|+4\rangle$ + 0.34346 $\|-4\rangle$<br>0.10707 $\|+3\rangle$ + 0.10707 $\|-3\rangle$<br>0.00610 $\|+2\rangle$ + 0.00610 $\|-2\rangle$<br>0.00034 $\|+1\rangle$ + 0.00034 $\|-1\rangle$<br>0.00093 $\|0\rangle$
3	0.00213 $\|+6\rangle$ + 0.00213 $\|-6\rangle$<br>0.04104 $\|+5\rangle$ + 0.04104 $\|-5\rangle$<br>0.35089 $\|+4\rangle$ + 0.35089 $\|-4\rangle$<br>0.09239 $\|+3\rangle$ + 0.09239 $\|-3\rangle$<br>0.01199 $\|+2\rangle$ + 0.01199 $\|-2\rangle$<br>0.00115 $\|+1\rangle$ + 0.00115 $\|-1\rangle$<br>0.00081 $\|0\rangle$
	0.000105 $\|+6\rangle$ + 0.000105 $\|-6\rangle$<br>0.005534 $\|+5\rangle$ + 0.005534 $\|-5\rangle$<br>0.088403 $\|+4\rangle$ + 0.088403 $\|-4\rangle$<br>0.256009 $\|+3\rangle$ + 0.256009 $\|-3\rangle$<br>0.124486 $\|+2\rangle$ + 0.124486 $\|-2\rangle$<br>0.024816 $\|+1\rangle$ + 0.024816 $\|-1\rangle$<br>0.001293 $\|0\rangle$
4	0.00011 $\|+6\rangle$ + 0.00011 $\|-6\rangle$<br>0.00580 $\|+5\rangle$ + 0.00580 $\|-5\rangle$<br>0.09277 $\|+4\rangle$ + 0.09277 $\|-4\rangle$<br>0.24661 $\|+3\rangle$ + 0.24661 $\|-3\rangle$<br>0.13603 $\|+2\rangle$ + 0.13603 $\|-2\rangle$<br>0.01673 $\|+1\rangle$ + 0.01673 $\|-1\rangle$

	$0.00390\ \lvert 0\rangle$
	$0.00001\ \lvert +6\rangle + 0.00001\ \lvert -6\rangle$<br>$0.00003\ \lvert +5\rangle + 0.00003\ \lvert -5\rangle$<br>$0.00494\ \lvert +4\rangle + 0.00494\ \lvert -4\rangle$<br>$0.13932\ \lvert +3\rangle + 0.13932\ \lvert -3\rangle$<br>$0.16448\ \lvert +2\rangle + 0.16448\ \lvert -2\rangle$<br>$0.12395\ \lvert +1\rangle + 0.12395\ \lvert -1\rangle$<br>$0.13454\ \lvert 0\rangle$
5	$0.000020\ \lvert +6\rangle + 0.000020\ \lvert -6\rangle$<br>$0.001442\ \lvert +5\rangle + 0.001442\ \lvert -5\rangle$<br>$0.020843\ \lvert +4\rangle + 0.020843\ \lvert -4\rangle$<br>$0.110195\ \lvert +3\rangle + 0.110195\ \lvert -3\rangle$<br>$0.160744\ \lvert +2\rangle + 0.160744\ \lvert -2\rangle$<br>$0.196569\ \lvert +1\rangle + 0.196569\ \lvert -1\rangle$<br>$0.020374\ \lvert 0\rangle$
	$0.00002\ \lvert +6\rangle + 0.00002\ \lvert -6\rangle$<br>$0.00032\ \lvert +5\rangle + 0.00032\ \lvert -5\rangle$<br>$0.00102\ \lvert +4\rangle + 0.00102\ \lvert -4\rangle$<br>$0.01963\ \lvert +3\rangle + 0.01963\ \lvert -3\rangle$<br>$0.11753\ \lvert +2\rangle + 0.11753\ \lvert -2\rangle$<br>$0.02040\ \lvert +1\rangle + 0.02040\ \lvert -1\rangle$<br>$0.68217\ \lvert 0\rangle$
6	$0.00006\ \lvert +6\rangle + 0.00006\ \lvert -6\rangle$<br>$0.00043\ \lvert +5\rangle + 0.00043\ \lvert -5\rangle$<br>$0.00091\ \lvert +4\rangle + 0.00091\ \lvert -4\rangle$<br>$0.02196\ \lvert +3\rangle + 0.02196\ \lvert -3\rangle$<br>$0.20113\ \lvert +2\rangle + 0.20113\ \lvert -2\rangle$<br>$0.25246\ \lvert +1\rangle + 0.25246\ \lvert -1\rangle$

	0.04607 |0⟩
7	0.000114 |+6⟩ + 0.000114 |−6⟩<br>0.000012 |+5⟩ + 0.000012 |−5⟩<br>0.000078 |+4⟩ + 0.000078 |−4⟩<br>0.004701 |+3⟩ + 0.004701 |−3⟩<br>0.077353 |+2⟩ + 0.077353 |−2⟩<br>0.363085 |+1⟩ + 0.363085 |−1⟩<br>0.109316 |0⟩

Table S7. Composition of energy states in **1**$_{wc}$ (given the small difference in the tunnelling gap compared to **1**, values reported to two decimal places for clarity).

**Energy state**	**Composition of the states**
1	0.49 |+6⟩ +0.49 |−6⟩
	0.49 |+6⟩ +0.49 |−6⟩
2	0.45 |+5⟩ + 0.05 |+4⟩ + 0.44 |−5⟩ + 0.05 |−4⟩
	0.45 |+5⟩ + 0.05 |+4⟩ + 0.45 |−5⟩ + 0.05 |−4⟩
3	0.04 |+5⟩ + 0.34 |+4⟩ + 0.10 |+3⟩ +0.04 |−5⟩ + 0.34 |−4⟩+ 0.10 |−3⟩
	0.04 |+5⟩ + 0.35 |+4⟩ + 0.09 |+3⟩ +0.04 |−5⟩ + 0.35 |−4⟩+ 0.04 |−3⟩
4	0.09 |+4⟩ + 0.26 |+3⟩ + 0.12 |+2⟩ + 0.02 |+1⟩ + 0.09 |−4⟩ + 0.26 |−3⟩ + 0.12 |−2⟩+ 0.02 |−1⟩
	0.09 |+4⟩ + 0.25 |+3⟩ + 0.14 |+2⟩ + 0.02 |+1⟩ + 0.09 |−4⟩ + 0.25 |−3⟩ + 0.14 |−2⟩+ 0.02 |−1⟩
5	0.14 |+3⟩ + 0.17 |+2⟩ + 0.13 |+1⟩ + 0.13 |0⟩ + 0.12 |−3⟩ + 0.16 |−2⟩ + 0.12 |−1⟩
	0.11 |+3⟩ + 0.16 |+2⟩ + 0.20 |+1⟩ + 0.02 |0⟩ + 0.11 |−3⟩ + 0.16 |−2⟩ + 0.20 |−1⟩
6	0.02 |+3⟩ + 0.11 |+2⟩ + 0.68 |0⟩ + 0.02 |−3⟩ + 0.12 |−2⟩
	0.01 |+3⟩ + 0.20 |+2⟩ + 0.25 |+1⟩ + 0.05 |0⟩ + 0.02 |−3⟩ + 0.20 |−2⟩ + 0.25 |−1⟩
7	0.08 |2⟩ + 0.36 |1⟩ + 0.11 |0⟩ + 0.36 |−1⟩ + 0.08 |−2⟩

Table S8. *Ab initio* SINGLE_ANISO computed crystal field parameters B_k^q of rank $k \leq 6$ associated with extended Stevens operators, $\hat{O}_k^q$ for **1** and **1**wc (without counter anion). Diagonal ($q = 0$) terms are highlighted in bold. Transverse terms for *hexagonal model 1* and *hexagonal model 2* (see main text) are highlighted in blue. Terms with $k \geq 8$ are given in Table S9.

k	*q*	B_k^q of 1 (cm^{-1})	B_k^q of 1wc (cm^{-1})
2	−2	−1.80×10^{-1}	6.86×10^{1}
2	−1	−1.10×10^{1}	8.26×10^{0}
**2**	**0**	**−9.67×10^{0}**	**−9.92×10^{0}**
2	1	2.31×10^{-1}	−6.88×10^{0}
2	2	7.16×10^{-1}	4.76×10^{-1}
4	−4	9.96×10^{-3}	−1.26×10^{-2}
4	−3	−3.20×10^{-2}	−8.68×10^{-3}
4	−2	−2.22×10^{-3}	−3.38×10^{-2}
4	−1	1.44×10^{-1}	−1.09×10^{-1}
**4**	**0**	2.14×10^{-2}	**2.20×10^{-2}**
4	1	−2.45×10^{-3}	9.02×10^{-2}
4	2	−3.57×10^{-2}	−6.50×10^{-3}
4	3	−5.67×10^{-3}	−2.84×10^{-2}
4	4	−9.08×10^{-3}	3.61×10^{-3}
6	−6	−8.15×10^{-4}	4.16×10^{-5}
6	−5	1.33×10^{-3}	1.71×10^{-3}
6	−4	1.33×10^{-4}	−1.60×10^{-4}
6	−3	1.90×10^{-4}	1.13×10^{-4}
6	−2	1.97×10^{-5}	3.57×10^{-4}
6	−1	−1.11×10^{-3}	8.53×10^{-4}
**6**	**0**	−3.30×10^{-5}	**−3.89×10^{-5}**
6	1	1.66×10^{-5}	−7.11×10^{-4}
6	2	3.73×10^{-4}	8.00×10^{-5}
6	3	−4.30×10^{-5}	1.39×10^{-4}
6	4	−1.14×10^{-4}	4.94×10^{-5}
6	5	1.87×10^{-3}	1.50×10^{-3}
6	6	6.13×10^{-4}	−1.05×10^{-3}

Hexagonal *model 1* for $k \leq 6$ (see main text): B_2^0, B_4^0, B_6^0 and $B_6^{\pm 6}$. Non-hexagonal *model 1* for $k \leq 6$ (see main text): all terms in the Table excluding $B_6^{\pm 6}$.

Table S9. *Ab initio* SINGLE_ANISO computed crystal field parameters B_k^q of rank $k \geq 8$ associated with extended Stevens operators, $\hat{O}_k^q$ for **1** and **1**wc (without counter anion). These terms, which are unphysical in a purely electrostatic picture, may arise due to covalency effects. Diagonal (q = 0) terms are highlighted in bold. Higher rank transverse terms for *hexagonal model 2* (see main text) are highlighted in blue.

k	***q***	B_k^q **of 1 (cm^{-1})**	B_k^q **of 1wc (cm^{-1})**
8	−8	4.27×10^{-07}	6.54×10^{-07}
8	−7	4.92×10^{-06}	7.23×10^{-06}
8	−6	2.32×10^{-06}	−1.56×10^{-07}
8	−5	−3.15×10^{-06}	−3.87×10^{-06}
8	−4	−1.28×10^{-06}	1.76×10^{-06}
8	−3	−4.98×10^{-06}	−3.61×10^{-06}
8	−2	−2.66×10^{-07}	−6.88×10^{-06}
8	−1	2.77×10^{-06}	−2.27×10^{-06}
**8**	**0**	**−1.75×10^{-07}**	**−1.60×10^{-07}**
8	1	−1.51×10^{-09}	1.77×10^{-06}
8	2	−7.17×10^{-06}	−1.89×10^{-06}
8	3	1.99×10^{-06}	−3.28×10^{-06}
8	4	1.49×10^{-06}	−8.13×10^{-07}
8	5	−7.48×10^{-06}	−7.73×10^{-06}
8	6	−1.75×10^{-06}	2.98×10^{-06}
8	7	6.91×10^{-06}	−4.84×10^{-06}
8	8	−5.56×10^{-07}	−6.95×10^{-08}
10	−10	5.25×10^{-09}	4.92×10^{-09}
10	−9	−4.55×10^{-10}	−2.29×10^{-09}
10	−8	−4.18×10^{-09}	−4.95×10^{-09}
10	−7	−3.15×10^{-08}	−4.38×10^{-08}
10	−6	−9.04×10^{-09}	−1.52×10^{-09}
10	−5	−1.79×10^{-08}	−2.44×10^{-08}
10	−4	−3.30×10^{-08}	3.58×10^{-08}
10	−3	4.95×10^{-08}	2.10×10^{-08}
10	−2	7.28×10^{-10}	1.49×10^{-08}
10	−1	−1.58×10^{-08}	1.85×10^{-08}
**10**	**0**	**4.89×10^{-09}**	**4.81×10^{-09}**
10	1	−1.63×10^{-09}	−8.16×10^{-09}
10	2	1.52×10^{-08}	6.07×10^{-09}
10	3	−3.97×10^{-09}	3.98×10^{-08}
10	4	1.43×10^{-08}	−4.20×10^{-09}
10	5	−1.82×10^{-08}	−1.47×10^{-08}
10	6	7.36×10^{-09}	−1.10×10^{-08}
10	7	−4.29×10^{-08}	3.21×10^{-08}
10	8	3.24×10^{-09}	−1.03×10^{-09}
10	9	−1.38×10^{-08}	1.22×10^{-08}
10	10	3.29×10^{-10}	−1.91×10^{-09}
12	−12	−2.35×10^{-10}	−1.03×10^{-11}
12	−11	−2.25×10^{-10}	−6.51×10^{-10}
12	−10	3.69×10^{-11}	3.89×10^{-11}

12	−9	7.23×10^{-11}	-5.15×10^{-11}
12	−8	-1.11×10^{-10}	-1.65×10^{-10}
12	−7	-5.14×10^{-10}	-7.91×10^{-10}
12	−6	-1.55×10^{-10}	1.33×10^{-11}
12	−5	3.02×10^{-10}	-2.37×10^{-10}
12	−4	1.32×10^{-10}	-1.61×10^{-10}
12	−3	-4.87×10^{-12}	-7.71×10^{-12}
12	−2	8.89×10^{-11}	2.04×10^{-11}
12	−1	-6.42×10^{-10}	2.83×10^{-10}
**12**	**0**	$\mathbf{-7.56\times10^{-11}}$	$\mathbf{-8.11\times10^{-11}}$
12	1	2.68×10^{-11}	-5.64×10^{-10}
12	2	-4.76×10^{-11}	-7.43×10^{-10}
12	3	3.23×10^{-11}	3.57×10^{-11}
12	4	-9.40×10^{-11}	4.12×10^{-11}
12	5	3.27×10^{-10}	7.51×10^{-10}
12	6	1.03×10^{-10}	-2.03×10^{-10}
12	7	-7.38×10^{-10}	5.21×10^{-10}
12	8	1.21×10^{-10}	-1.37×10^{-11}
12	9	7.68×10^{-11}	-8.44×10^{-11}
12	10	1.90×10^{-11}	1.51×10^{-12}
12	11	8.37×10^{-10}	-5.55×10^{-10}
12	12	-6.48×10^{-11}	2.48×10^{-10}

Hexagonal *model 2* for $k \leq 12$ (see main text): B_2^0, B_4^0, B_6^0, B_8^0, B_{10}^0, B_{12}^0, $B_6^{\pm6}$, $B_8^{\pm6}$, $B_{10}^{\pm6}$, $B_{12}^{\pm6}$ and $B_{12}^{\pm12}$. Non-hexagonal *model 2* for $k \leq 12$ (see main text): all terms in the Table excluding $B_6^{\pm6}$, $B_8^{\pm6}$, $B_{10}^{\pm6}$, $B_{12}^{\pm6}$ and $B_{12}^{\pm12}$.

Table S10. Effects on the calculated tunnel splitting in **1** and **1**$_{\mathrm{wc}}$ (without counter anion) based on symmetry-restricted models.

**Model**	**Tunnel splitting of 1 (GHz)**	**Tunnel splitting of 1**$_{\mathbf{WC}}$ **(GHz)**
All allowed $k = 2$ terms	0	0
All allowed $k = 2, 4$ terms	0.02	0.03
All allowed $k = 2, 4, 6$ terms	5.75	5.26
All allowed $k = 2, 4, 6, 8$ terms	3.78	3.46
All allowed $k = 2, 4, 6, 8, 10$ terms	4.35	4.17
Full Hamiltonian (all allowed $k \leq 12$ terms)	0.84	0.92
Ab initio	**0.84**	**0.92**

EPR Analysis

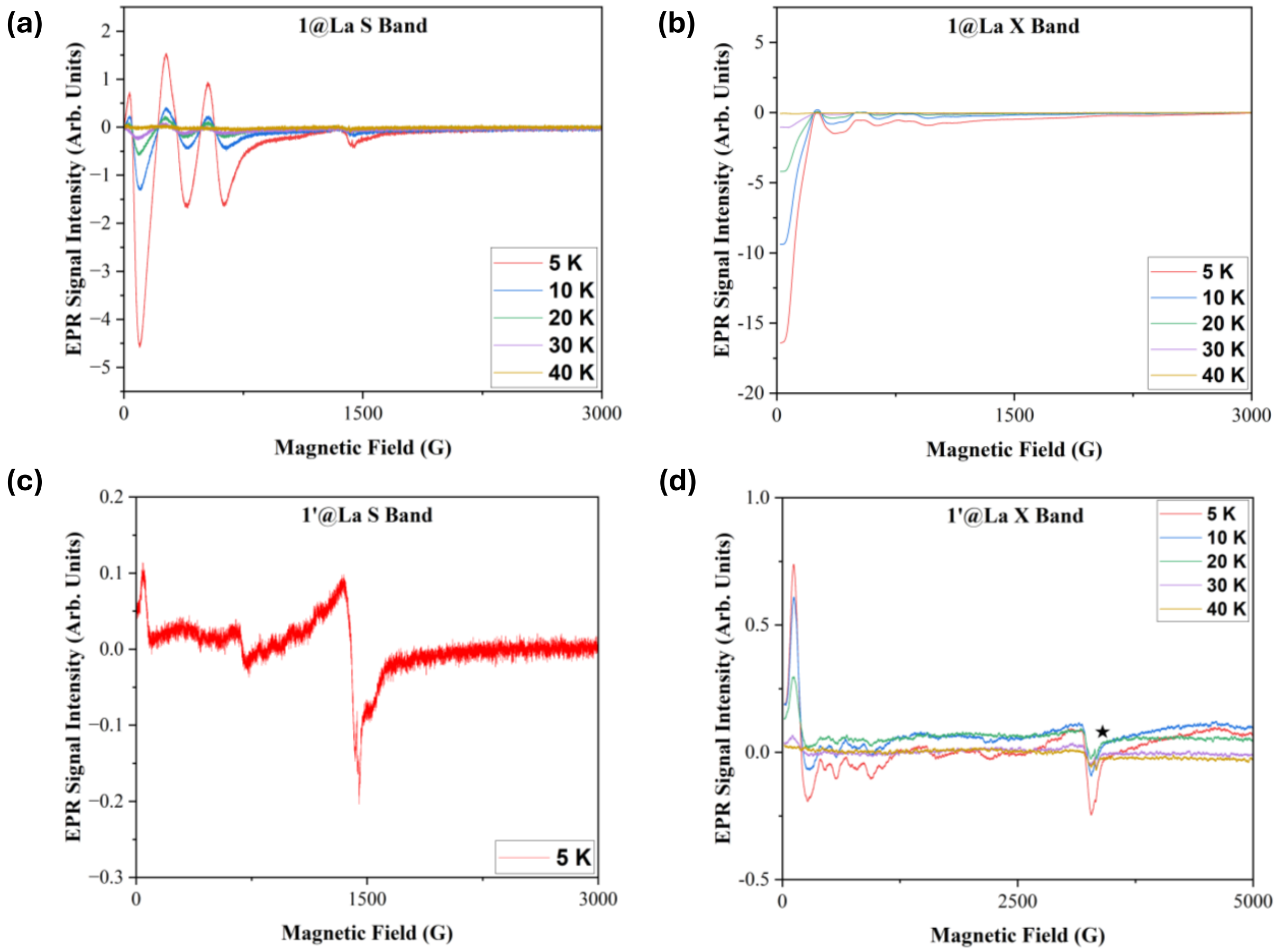

Figure S16. (a) Temperature dependence of the S-band EPR spectrum of **1@La** as 6% Tb, (b) temperature dependence of the X-band EPR spectrum of **1@La** as 6% Tb, (c) S-band EPR spectrum recorded at 5 K for **1'@La** as 0.7% Tb and (d) temperature dependence of X-band EPR spectra of **1'@La** as 0.7% Tb. The star indicates the Cu(II) background signal from the resonator.

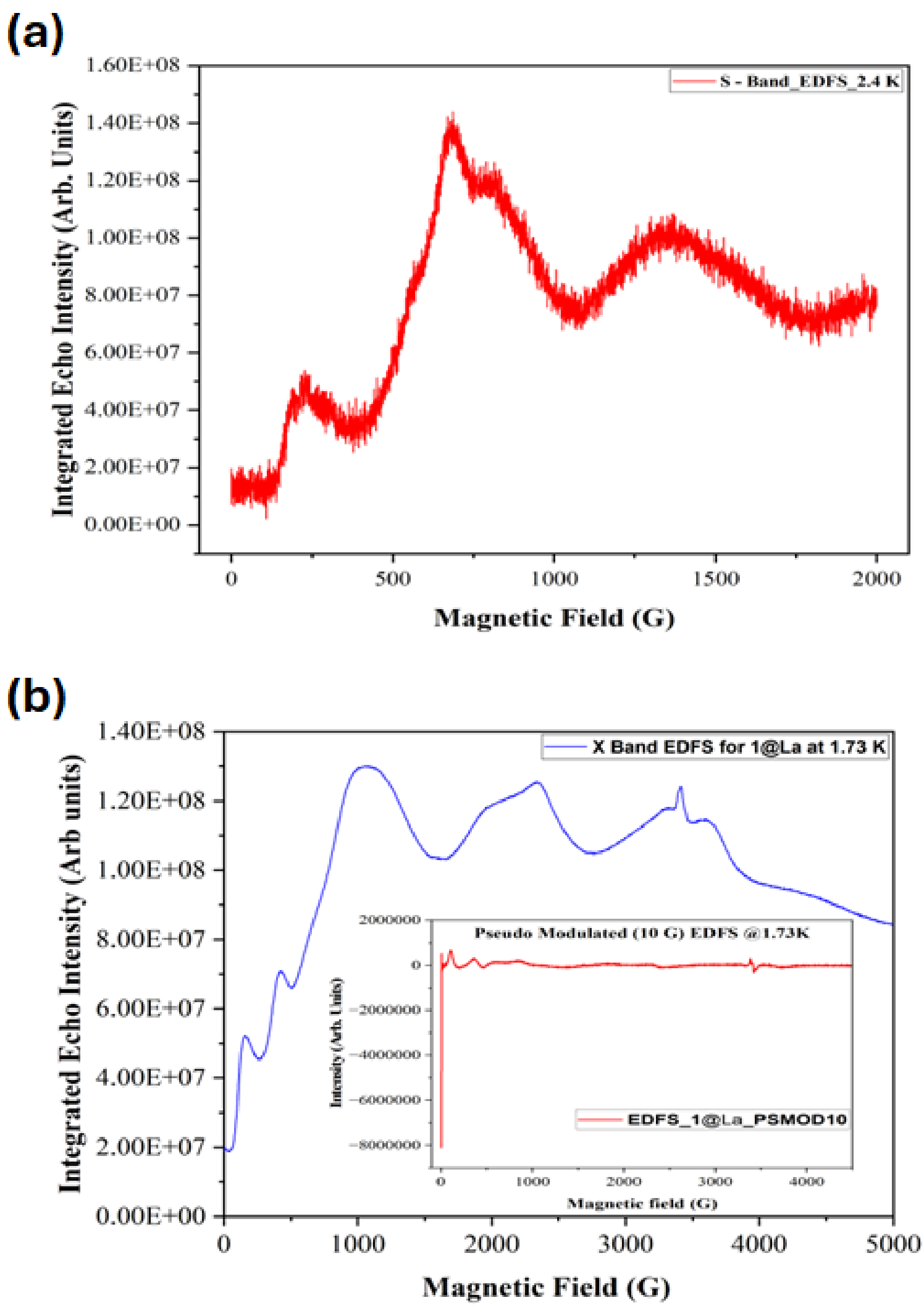

Figure S17. (a) Echo-detected field sweep (EDFS) spectrum recorded at S-band for **1@La** at 2.4 K and (b) EDFS spectrum recorded at X-band for **1@La** at 1.73 K. Measurements were performed on powdered crystals, rather than single crystals, to enhance the signal-to-noise (S/N) ratio. Multiple parameter sets were tested to avoid losing echoes through positional or saturation effects. Final data were collected under maximized spectrometer conditions: lowest temperature, fastest shot repetition rate, and the highest feasible number of averages.

Echo-detected field sweeps (EDFS) were carried out for **1@La** (see Figure S17), however, the echo-detected EPR signals from the Tb(III) complex were exceedingly weak and could only be captured under highly optimized conditions. These required extensive signal averaging, short repetition times, and operation at the lower temperature limits of the spectrometer capabilities. Among the frequencies tested, X-band measurements provided the closest correspondence to the CW spectra reported by Harris and co-workers for Tb(III) glasses.[2] Notably, the *pseudo*-modulated pulsed response (Figure S17b inset) in our study bore resemblance to the CW signal, which extends up to ~1000 G (Figure 4). The background cavity signal near 3500 G was used to validate the phase of the measured echo and ensure instrumental consistency. The absence of a pronounced spin echo is most plausibly attributed to a

combination of fast relaxation time and a relatively low number of resonant spins (see Table S6, S7) in the powder sample. Unlike the frozen solution methodology employed by Eaton *et al.,*[3] which affords better control over spin concentration and homogeneity, our system is limited to modulation via dilution levels in the solid state. This constraint likely contributes to diminished signal intensities and increased relaxation driven decoherence.

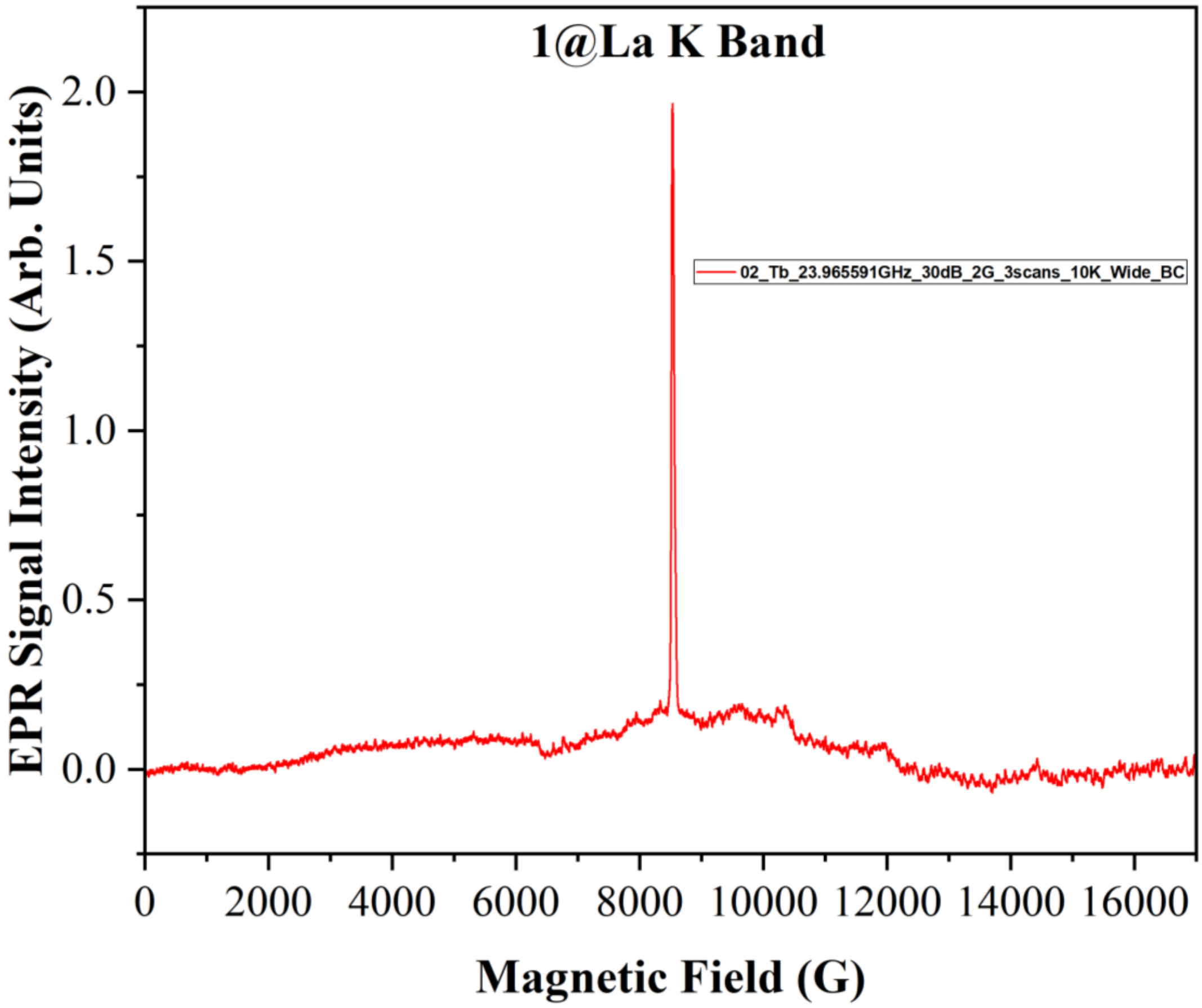

Figure S18. Field swept K band spectra for **1@La** as 6% Tb recorded at 10 K. The feature observed here corresponds to no meaningful signal and is instead attributed to a defect in the tube glassware, appearing inverted due to saturation. Its sole relevance is as a reference marker at g ≈ 2, included to demonstrate the absence of any reliable K-band transitions.

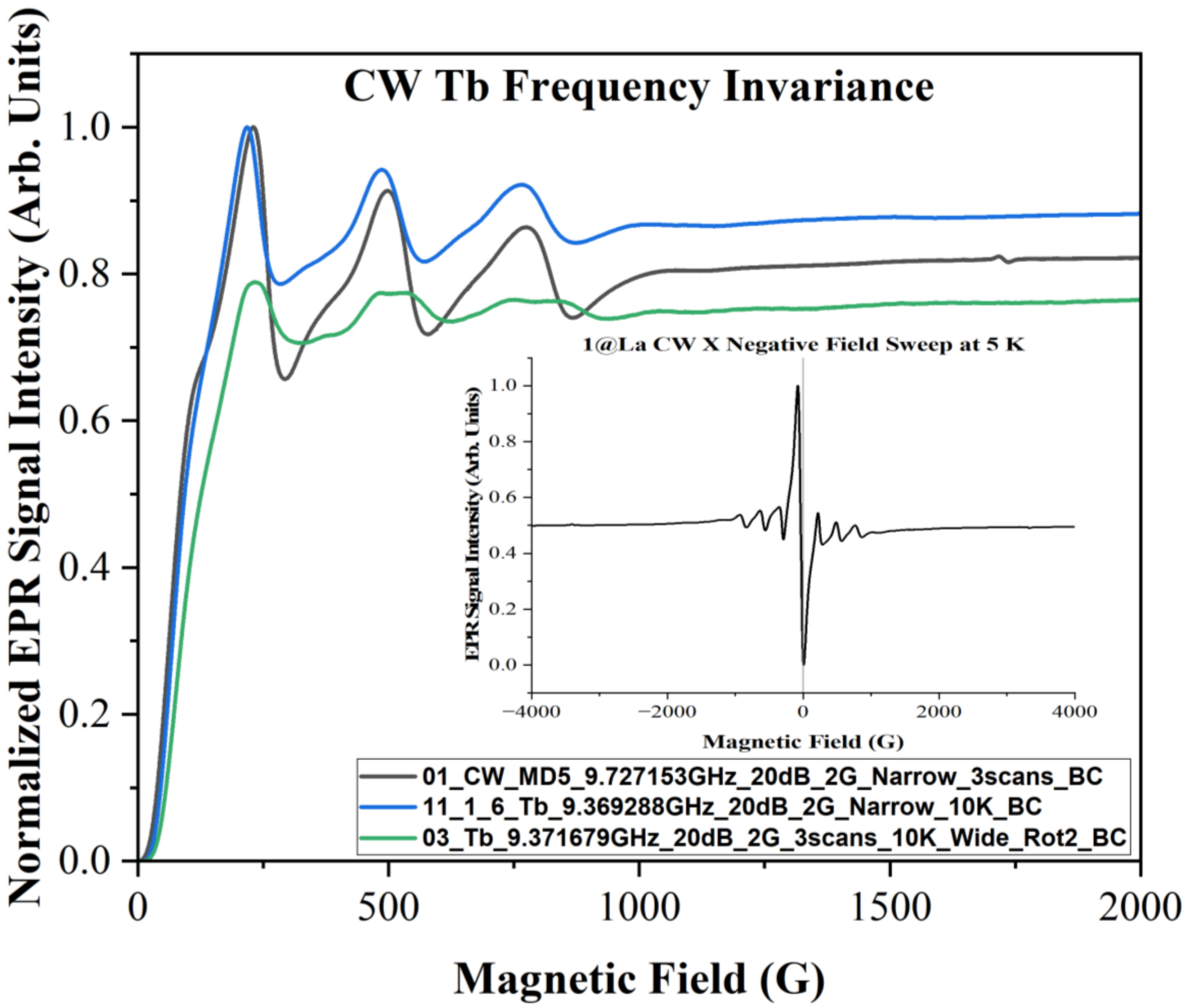

Figure S19. X-band frequency variance of **1@La** as 6% Tb, inset: Field swept spectra across negative to positive values, depicting symmetry of spectrum about zero field.

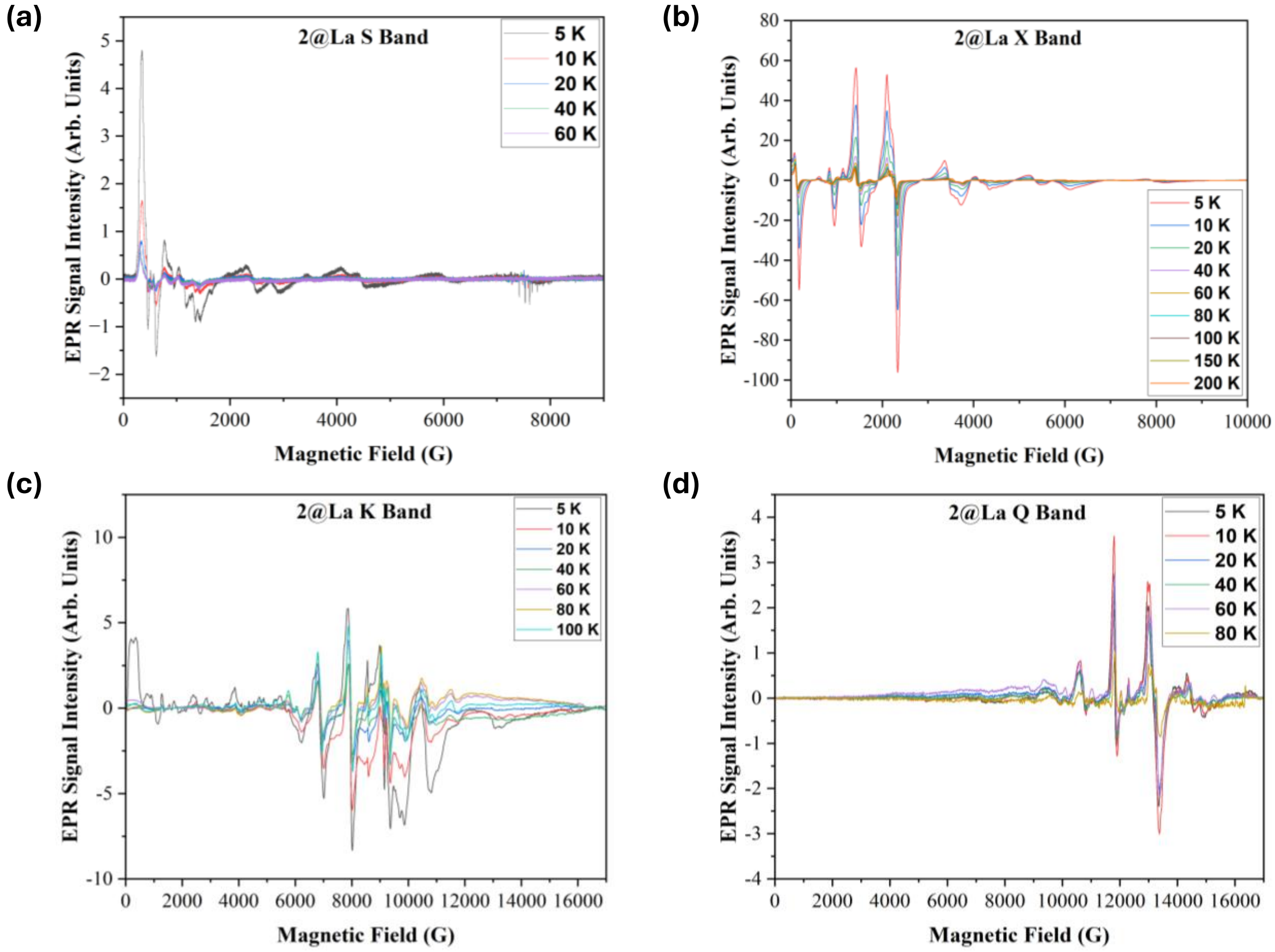

Figure S20. Multifrequency variable temperature continuous wave EPR spectra of **2@La** as 8.8% Gd recorded at (a) S-band, (b) X-band, (c) K-band and (d) Q-band.

References

[1] Canaj, A. B.; Dey, S.; Martí, E. R.; Wilson, C.; Rajaraman, G.; Murrie, M. Insight into D_{6h} symmetry: Targeting strong axiality in stable Dysprosium(III) hexagonal bipyramidal single-ion magnets. *Angew. Chem. Int. Ed.* **2019**, *58* (40), 14146—14151. DOI: 10.1002/anie.201907686

[2] Harris, E. A.; Furniss, D. Electron paramagnetic resonance of terbium in a zirconium fluoride glass. *J. Phys. C. Solid State* **1988**, *21* (1), 7—15. DOI: 10.1088/0022-3719/21/1/004

[3] McPeak, J.; Alexander, D.; Joseph, C.; Eaton, S. S.; Eaton, G. R. Electron spin relaxation of Tb^{3+} and Tm^{3+} ions. *Appl. Magn. Reson.* **2020**, *51* (9-10), 961—976. DOI: 10.1007/s00723-020-01262-6
